# Synthesis and Application of Bioactive *N*‐Functionalized Aziridines

**DOI:** 10.1002/anie.202514630

**Published:** 2025-08-22

**Authors:** Hao Tan, Samya Samanta, Nan Qiu, Alexander Adibekian, David C. Powers

**Affiliations:** ^1^ Department of Chemistry Texas A&M University College Station TX 77843 USA; ^2^ Department of Chemistry University of Illinois Chicago Chicago IL 60607 USA; ^3^ Skaggs Graduate School and Chemical and Biological Sciences Scripps Research La Jolla CA 92037 USA; ^4^ Department of Pharmaceutical Sciences University of Illinois Chicago Chicago IL 60612 USA; ^5^ Department of Biochemistry and Molecular Genetics University of Illinois Chicago Chicago IL 60607 USA; ^6^ University of Illinois Cancer Center Chicago IL 60607 USA; ^7^ UICentre University of Illinois Chicago Chicago IL 60612 USA

**Keywords:** Aziridines, Bioactive small molecules, Catalysis, Lipidomics, Proteomics

## Abstract

Aziridines—three‐membered nitrogen heterocycles that engage in strain‐accelerated ring opening chemistry—are not common functional groups in bioactive natural products. As such, the discovery and optimization of predictable and general methods for the construction of aziridines is critical to the development, evaluation, and optimization of small molecules that contain aziridines. In this review, we discuss modern synthetic strategies for the construction of aziridines. Synthetic methods are categorized based on the synthetic logic used to assemble the aziridine ring: 1) Addition of nitrene equivalents to olefins, 2) addition of carbene equivalents to imines, and 3) intramolecular cyclization chemistry. Special emphasis is given to methods that allow modular control over the identity of the exocyclic nitrogen valence, which directly impacts the electrophilicity, and thus biological activity, of the resulting aziridines. After describing the state of the art in aziridine synthesis, we discuss established and emerging biological applications of aziridine‐containing small molecules, including application as proteomics probes that enable liganding of nonstandard amino acid residues and application in lipidomics. Finally, extant synthetic challenges that must be addressed to realize the full potential of aziridine‐based small molecules are described.

## Graphical abstract

This review discusses modern synthetic methods for the preparation of aziridine‐containing small molecules, including biocatalytic, electrocatalytic, and photocatalytic strategies. We highlight the compatibility of various synthetic methods with control of the exocyclic *N‐*substituent. These methods enable application of aziridines as activity‐based probes for glycosidases, drug development, proteomics, and lipidomics.

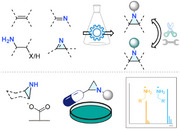

## 1 Introduction

**Figure 1:**
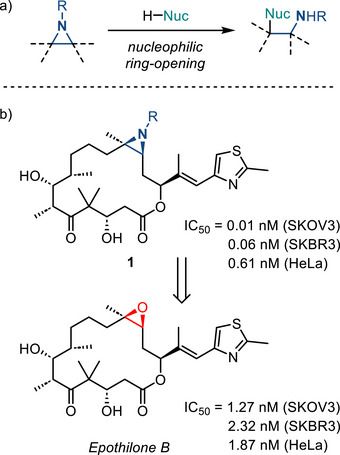
a) Aziridines engage in ring‐opening chemistry with a wide variety of nucleophiles. b) Compound **1**, the aziridine analogue of Epothilone B, displays significantly increased potency against a variety of cell lines.

Aziridines are the smallest saturated nitrogen‐containing heterocycles and engage in strain‐accelerated ring‐opening chemistry with nucleophiles (Figure 1a).^[^ ^1^ ^]^ Aziridines are not often encountered in natural products: While robust enzymatic machinery is available to construct epoxides, which are the oxygen analogues of aziridines, from olefinic precursors, analogous aziridination chemistry has not naturally evolved.^[^ ^2^ ^]^ Despite the relative dearth of naturally occurring aziridines, they represent attractive synthetic targets because 1) aziridines represent the reactive site (i.e., chemical warhead) in a growing number of biologically active small molecules,^[^ ^3^, ^4^, ^5^ ^]^ and 2) predictable ring‐opening chemistry provides a modular strategy to rapidly access 1,2‐aminofunctionalization products from aziridine precursors.^[^ ^6^, ^7^ ^]^

Aziridination can have a profound impact on biological activity of active pharmaceutical ingredients (APIs). For example, aziridine **1**, the nitrogen analogue of epothilone B, displays marked improvement in pharmacological activity (Figure 1b).^[^ ^8^ ^]^ The enhanced activity of aziridine‐containing APIs is typically ascribed to covalent inhibition mechanisms: Reaction of small molecule aziridines with biological nucleophiles provides a mechanism for covalent liganding of biologically relevant sites.^[^ ^8^ ^]^ From a molecular design perspective, the electrophilicity of aziridines can be tuned by systematic variation of the exocyclic *N*‐substituents, which enables more comprehensive structure–activity relationship (SAR) screening than the corresponding epoxides.^[^ ^9^ ^]^ As such, the development of new strategies to introduce aziridine rings into small molecule scaffolds and methods that enable control over the identity of the exocyclic *N*‐valence are critical to realizing the potential of aziridine‐based small molecules.

In this review, we describe advances in the synthetic chemistry of aziridines with particular focus on methods that have been developed since 2015. The interested reader is directed to reviews and monographs that emphasize other aspects of the synthesis and application of aziridine‐containing small molecules.^[^ ^4^, ^10^, ^11^ ^]^ The discussion of aziridines synthesis is organized by the bond disconnections used to assemble the three‐membered ring: 1) Addition of nitrene equivalents to olefins, 2) addition of carbene equivalents to imines, and 3) intramolecular cyclization of β‐functionalized amines, including recent progress in β‐C─H activation of aliphatic amines. For each disconnection, opportunities to derivatize of the exocyclic *N*‐valence will be discussed. In particular, we discuss strategies to 1) access N─H aziridines, 2) functionalize N─H aziridines, 3) accomplish aziridine cross‐coupling, and 4) effect aziridine group transfer chemistry. After discussing the state‐of‐the‐art in aziridine synthesis, emerging applications of aziridine‐containing small molecules in medicinal chemistry, proteomics, and lipidomics will be discussed. Emphasis will be given to the relationship between synthetic methods employed and the applications that are thus enabled. Finally, continuing challenges for both synthetic chemistry and the biological application of aziridine‐based small molecules will be discussed.

## 2 Synthesis of Aziridines

**Figure 2:**
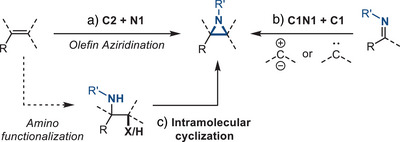
General strategies to access aziridines include a) addition of nitrene equivalents to olefins, b) addition of carbene equivalents to imines, and c) intramolecular cyclization.

Three major strategies have emerged for the synthesis of aziridines: 1) Addition of nitrene equivalents to olefins (N1 + C2), 2) addition of carbene equivalents to imines (C1 + N1C1), and 3) intramolecular cyclization of β‐functionalized amines (Figure 2). In this section, we discuss recent advances via each of these disconnections as well as emerging reactions that demonstrate alternate aziridine disconnections. The reaction mechanism of each transformation dictates the substrate scope. In the following discussion, we refer to substrates in which the olefin is conjugated with other functional groups (e.g., R = aryl or carbonyl) to be “activated,” while substrates in which the olefin is isolated from conjugation to be “unactivated.” For each method, the substrate scope and limitations with respect to the identity of the exocyclic *N*‐substituents will be discussed.

### 2.1 Olefin Aziridination

The widespread availability and natural abundance of olefin‐containing small molecules has motivated extensive efforts to develop efficient and selective nitrene group‐transfer chemistry and catalysis with olefinic substrates. From a historical perspective, in 1991, Evans reported a Cu‐catalyzed olefin aziridination method using PhINTs.^[^ ^12^ ^]^ Following this seminal report, myriad transition metal‐catalyzed methods have been developed for nitrene transfer to olefins.^[^ ^13^, ^14^ ^]^ More recently, a variety of (N1 + C2)‐type olefin aziridinations have been discovered with enzymatic catalysis and single‐atom catalysis. Metal‐free approaches have also been developed for olefin aziridination, typically utilizing electrophilic or ambiphilic aminating reagents. Additionally, photochemistry and electrochemistry have emerged as key tools for (N1 + C2)‐type aziridination.

#### 2.1.1 Metal‐Catalyzed Olefin Aziridination

The direct intermolecular transfer of simple organic nitrenes to olefins to construct *N*‐functionalized aziridines is typically not possible due to the short lifetimes of unstabilized nitrenes: Aliphatic nitrenes rearrange to imines, and aromatic nitrenes engage in ring‐expansion chemistry faster than addition to olefinic partners.^[^ ^15^, ^16^ ^]^ To overcome these challenges, families of *N*‐substituents, such as *N*‐sulfonyl, *N*‐sulfamoyl, and *N*‐acyl derivatives have been developed, and various catalysts that control the reactivity and selectivity of nitrene transfer reactions have been developed.^[^ ^17^, ^18^, ^19^ ^]^ These reactions are predicated on the electrophilic addition of a reactive metal nitrene intermediate to an olefinic substrate. Breslow and Evans reported some of the earliest metal‐catalyzed nitrene transfer reactions and early contributions largely focused on the development of reactions that afforded *N*‐sulfonyl aziridines. The *N*‐sulfonyl substituent is useful because it enhances the electrophilicity of incipient metal nitrene intermediates while suppressing *N*‐centered oxidation during the generation of nitrene equivalents. Early efforts provided a rich array of catalysts and reagents for nitrene transfer, with Rh_2_‐catalyzed methods being prominent among these initial studies. The resulting metal‐catalyzed reactions typically afforded *N*‐sulfonyl aziridines, or related *N*‐functionalized compounds. To access generic *N*‐functionalized aziridines, the *N*‐activating group must then be removed before functionalization can be pursued.

**Figure 3:**
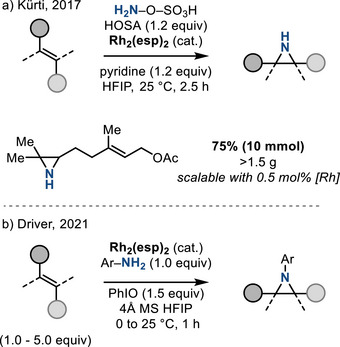
Rh_2_‐catalyzed olefin aziridination to access a) N─H aziridines with hydroxylamine‐*O*‐sulfonic acid (HOSA), and b) *N*‐aryl aziridines with anilines and iodosylbenzene.

In 2014, Jat et al. reported a stereospecific Rh_2_‐catalyzed aziridination of olefins with *O*‐(2,4‐dinitrophenyl)hydroxylamine (DPH) as nitrene source and 2,2,2‐trifluoro‐ethanol (TFE) as the solvent to construct N─H aziridines from both unactivated (i.e., aliphatic) and activated (i.e., styrenyl) olefins.^[^ ^20^ ^]^ The same investigators subsequently demonstrated analogous aziridination using hydroxylamine‐*O*‐sulfonic acid (HOSA), which is a more practical aminating reagent (Figure 3a).^[^ ^21^ ^]^ This method could be scaled up to 10 mmol, which highlights the preparative utility of the method. These methods were notable because metal‐catalyzed syntheses of aziridines bearing an unfunctionalized N─H valence are rare.

In 2021, Driver disclosed a Rh_2_‐catalyzed *N‐*arylaziridination reaction by combining anilines with iodosylbenzene (PhIO) under the action of Rh_2_(esp)_2_ catalysis (Figure 3b).^[^ ^22^ ^]^ This method, which is efficient for the aziridination of internal unactivated alkenes, achieves aziridination in preference to arylnitrene ring expansion, which is often sufficiently rapid as to prevent intermolecular nitrene transfer. The developed *N‐*arylaziridination was limited to electron‐deficient anilines, presumably to avoid competing *N*‐oxidation.

**Figure 4:**
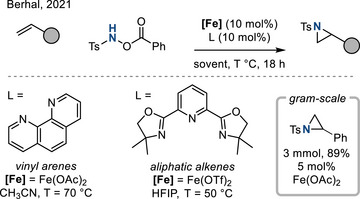
Fe‐catalyzed aziridination of both vinyl arenes and aliphatic alkenes.

As part of efforts to develop aziridination chemistry with inexpensive, earth‐abundant metal catalysts, in 2021, Berhal developed the iron‐catalyzed aziridination of activated and unactivated olefins using hydroxylamine derivative **2** as both the nitrene precursor and the oxidant.^[^ ^23^ ^]^ Styrene derivatives undergo aziridination in presence of Fe(OAc)_2_ and 1,10‐phenanthroline; for the relatively more challenging aliphatic olefins, Fe(OTf)_2_ in combination with a tridentate pyridine (bisoxazoline) (PyBOX) ligand was optimal. The method could be carried out on gram scale with styrene as the olefinic substrate (89% yield with 5 mol% catalyst). Deuterium labeling experiments provided evidence of stereospecific aziridination, which was interpreted as evidence of concerted [2 + 1] cycloaddition between transient iron nitrenes and the olefinic substrates (Figure 4).

**Figure 5:**
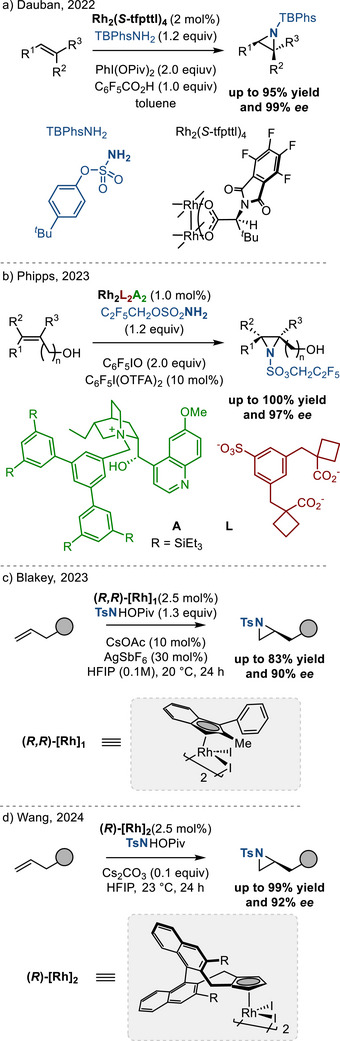
Enantioselective intermolecular olefin aziridination with iminoiodinane reagents a) and b), and hydroxylamine derivatives c) and d) by chiral Rh catalysis to access *N*‐sulfonyl aziridines.

Significant progress has been made toward asymmetric metal‐catalyzed olefin aziridination using iminoiodinanes as nitrene sources (Figure 5). In 2022, Dauban reported the enantioselective intermolecular olefin aziridination using chiral C_4_ symmetrical rhodium catalyst, achieving high yields and enantioselectivity (up to 99% ee; Figure 5a).^[^ ^24^ ^]^ In 2023, Phipps reported enantioselective aziridination of alkenyl alcohols using cinchona alkaloid‐derivatized chiral cations and chiral recognition based on non‐covalent, Coulombic interactions (Figure 5b).^[^ ^25^ ^]^ In 2023, Blakey, and in 2024, Wang independently developed enantioselective olefin aziridination reactions with hydroxylamines and unactivated olefins promoted by chiral cyclopentadienyl‐rhodium(III) catalysts (Figure 5c,d).^[^ ^26^, ^27^ ^]^

**Figure 6:**
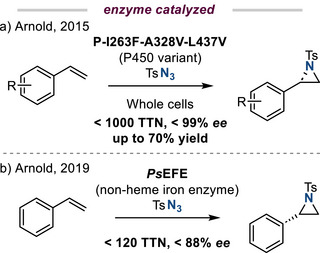
Enzyme‐catalyzed olefin aziridination can be achieved with both a) iron‐heme and b) nonheme iron enzymes. Directed evolution provides the opportunity to optimize catalyst performance in these reactions.

*Biocatalysis*. In 2015, Arnold reported an enantioselective styrene aziridination with tosyl azide using an engineered cytochrome P450 catalyst (Figure 6a).^[^ ^28^ ^]^ The optimized biocatalyst displayed a turnover number (TON) greater than 1000 and effected aziridination in excellent enantioselectivity (>99% ee). In 2019, the same group demonstrated *Pseudomonas savastanoi* ethylene‐forming enzyme (psEFE), a nonheme iron enzyme, can also catalyze styrene aziridination.^[^ ^29^ ^]^ Directed evolution of this enzyme provided the opportunity to improve catalyst performance in terms of both TON and stereocontrol (Figure 6b).^[^ ^29^ ^]^

**Figure 7:**
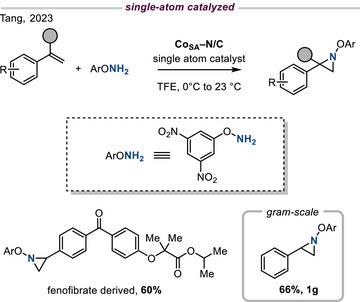
Single‐atom catalysis (SAC) for aziridination. Co─N/C promotes aziridination of styrene derivatives using hydroxylamine derivatives as the nitrogen source.

Single‐atom catalysts (SACs) have garnered significant contemporary interest as platforms for heterogeneous catalysis and have recently been applied to olefin aziridination. Specifically, a Co─N/C catalyst derived from carbonization of a Co imidazolate framework exhibits efficient olefin aziridination activity using hydroxylamines as the nitrogen source.^[^ ^30^ ^]^ The observed reactivity was ascribed to atomically dispersed Co atoms within the catalyst materials. The method was compatible with late‐stage functionalization of olefins derived from bioactive small molecules and gram‐scale synthesis, which demonstrates the synthetic potential of single‐atom catalysts (Figure 7).

#### 2.1.2 Transition Metal‐Free Aziridination

Concurrent to the development of metal‐catalyzed aziridine syntheses, a family of metal‐free methodologies has also been disclosed. The development of metal‐free methods is motivated by sustainability and cost considerations as well as the promise that these methods may offer complementary aziridination substrate scope and selectivity to available metal‐catalyzed processes.

**Figure 8:**
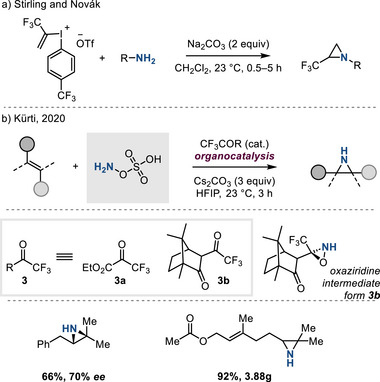
Metal‐free olefin aziridination has been developed with a) vinyl iodonium salts and amines and b) organocatalysis via oxaziridine intermediates.

In 2018, Stirling and Novák disclosed a base‐promoted aziridination protocol that combines amines and alkenyl iodonium reagents.^[^ ^31^ ^]^ A newly designed bench‐stable trifluoromethylated hypervalent alkenyl iodonium species—a C_2_─CF_3_ synthon—can exploit nucleophilic amines for this aziridine synthesis in presence of base under mild conditions (Figure 8a).

In 2020, Kürti described the aziridination of unactivated olefins using HOSA in the presence of electron‐deficient ketones, i.e., **3**, as catalysts (Figure 8b).^[^ ^32^ ^]^ An oxaziridine intermediate, generated by in situ reaction of HOSA with ketone **3**, was proposed to transfer nitrogen group via a concerted reaction with olefins. Chemoselective aziridination to unactivated C═C bonds over activated C═C bonds was observed and encouraging levels of enantioinduction (i.e., up to 70% ee with **3b**) were obtained. This method complements metal‐catalyzed methods for the synthesis of N─H aziridines by providing both a metal‐free catalyst and enabling enantioselective aziridine synthesis.^[^ ^20^ ^]^

**Figure 9:**
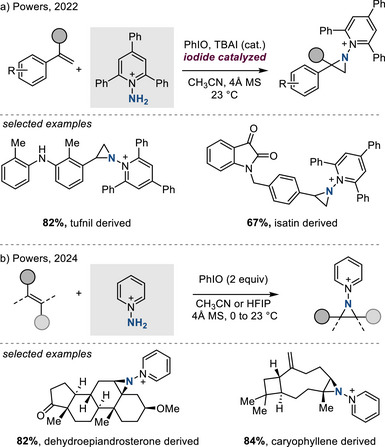
Metal‐free *N‐*pyridiniumaziridination of a) styrenes and b) aliphatic olefins.

In 2022, Powers introduced *N*‐aminopyridinium salts as a nitrogen source for metal‐free aziridination of styrenyl olefins in the presence of PhIO under the action of iodide catalysis (Figure 9a).^[^ ^33^ ^]^ This method can be applied to complex molecules and is thus compatible with late‐stage aziridination. In 2024, the same authors extended these studies to the aziridination of aliphatic olefins by accessing *N*‐pyridinium iminoiodinane intermediates, which are more electrophilic than traditional iminoiodinanes by virtue of the positively charged *N*‐pyridinium substituent (Figure 9b).^[^ ^34^ ^]^ These methods were developed due to the ease of functionalization of the N─N bond in *N*‐pyridinium aziridines, which provides a handle for *N*‐centered derivatization (see Section 3.2).^[^ ^7^, ^35^, ^36^ ^]^

**Figure 10:**
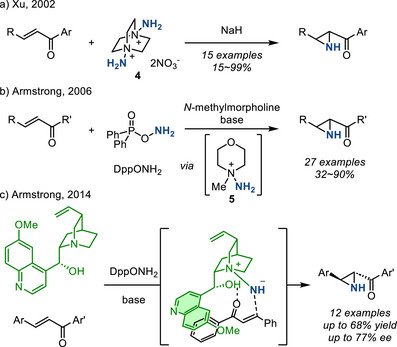
Aziridination of chalcone derivatives via hydrazinium salts.

*Aziridination of Electron‐Deficient Olefins*. Electron‐deficient C═C double bonds are challenging substrates for the electrophilic nitrene transfer reactions described above. Ambiphilic nitrogen sources, which combine *N*‐centered nucleophilicity with an appropriate N─X leaving group and thus can engage in aza‐Michael induced ring closure (aza‐MIRC) processes, have been developed to accomplish aziridination of Michael acceptors. The earliest demonstration of this strategy was reported in 2002 by Xu, who utilized *N*,*N*′‐diamino‐1,4‐diazoniabicyclo[2.2.2]octane dinitrate (**4**) in the presence of NaH for the aziridination of α,β‐unsaturated ketones to form the corresponding N─H aziridines (Figure 10a).^[^ ^37^ ^]^ Later, in a series of reports by Armstrong, *N*‐methylmorpholinium salt **5** was used as the nitrogen source for aziridination. *N*‐methylmorpholinium salt **5** could be generated in situ using *O*‐(diphenylphosphinyl)hydroxylamine (DppONH_2_) as the terminal nitrogen source (Figure 10b).^[^ ^38^, ^39^, ^40^, ^41^ ^]^ Notably, use of cinchona alkaloid‐derived mediators enabled asymmetric aza‐MIRC to access chiral N─H aziridines (Figure 10c).^[^ ^42^ ^]^ At present, this method requires stoichiometric loading of cinchona alkaloids. Methods using other electrophilic aminating reagents for enantioselective aziridination of α,β‐unsaturated carbonyl compounds with chiral organocatalysts have also been reported.^[^ ^43^, ^44^, ^45^, ^46^, ^47^ ^]^

**Figure 11:**
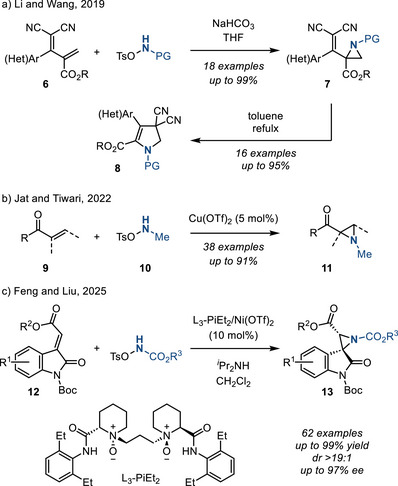
Aziridination of electron‐deficient alkenes with *O*‐tosylhydroxylamines can afford a) *N*‐protected aziridines that participate in subsequent ring‐expansion chemistry (PG = ─Boc, ─CO_2_Et, ─Cbz, and ─Ts), b) *N*‐methyl aziridines, and c) *N*‐protected aziridine isatin derivatives.

*O‐*Tosylhydroxylamines are commonly used reagents for the aziridination of electron‐deficient olefins. In 2019, Li and Wang reported chemoselective aziridination of alkenes **6** to afford *N*‐protected aziridines **7** (Figure 11a). The isolated aziridines engaged in subsequent ring expansion via a thermally promoted intramolecular rearrangement to form 2‐pyrrolines **8**.^[^ ^48^ ^]^ In 2022, Jat and Tiwari reported aziridination of chalcone derivatives **9** with hydroxylamine‐*O*‐sulfonates **10** under the action of Cu(II) catalysis to afford *N*‐methyl aziridines **11** in good yields and diastereoselectivity (dr up to 99:1) (Figure 11b).^[^ ^49^, ^50^ ^]^ In 2025, Feng and Liu reported the enantioselective aziridination of isatin derivatives **12** via chiral Lewis acid catalysis to afford spiro aziridine products **13** in good yields and ee (Figure 11c).^[^ ^51^ ^]^

#### 2.1.3 Photochemical Aziridination

**Figure 12:**
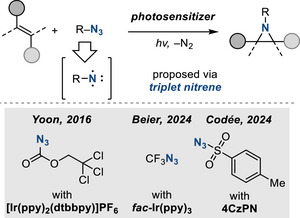
Photochemical olefin aziridination with organoazides under the action of triplet sensitizers.

Photochemical activation of electrophilic aminating reagents can provide complementary methods for olefin aziridination. A family of photochemical aziridination reactions has been developed by activation of organic azide precursors (Figure 12). Direct photolysis of organic azides effects N_2_ elimination and the generation of organic nitrenes. For simple organic azides, nitrenes are initially formed as singlets that then relax to the lower‐energy triplet configuration. In general, achieving selectivity between olefin aziridination and allylic amination products (as well as competing unimolecular rearrangement processes of transient nitrene intermediates) is a significant challenge. In some cases, these challenges have been overcome and have enabled synthetically useful photochemical nitrene transfer chemistry.

The use of appropriate triplet sensitizers can enable nitrene synthesis under visible‐light irradiation. In 2016, Yoon reported visible‐light‐promoted olefin aziridination with azidoformate‐based nitrene precursors in the presence of Ir‐based triplet sensitizers.^[^ ^52^ ^]^ The observed aziridination reactivity was ascribed to the intermediacy of transient triplet nitrenes, generated in a spin‐selective photoreaction. Both activated and unactivated olefins take part in this aziridination protocol.

Application of similar triplet sensitization has enabled the development of related olefin aziridination methods. In 2024, Beier reported the synthesis of *N*‐trifluoromethyl aziridines using trifluoromethyl azide via a triplet trifluoromethyl nitrene intermediate that was photogenerated with Ir(ppy)_3_ as the sensitizer.^[^ ^53^ ^]^ Also in 2024, Codée and Zhu described an analogous metal‐free reaction that utilized sulfonyl azides as nitrene precursors and employed organic photosensitizers, i.e., 3,4,5,6‐tetra(9*H*‐carbazol‐9‐yl)phthalonitrile (4CzPN) and 2,4,5,6‐tetrakis(diphenylamino)isophthalonitrile (4DPAIPN).^[^ ^54^, ^55^ ^]^

**Figure 13:**
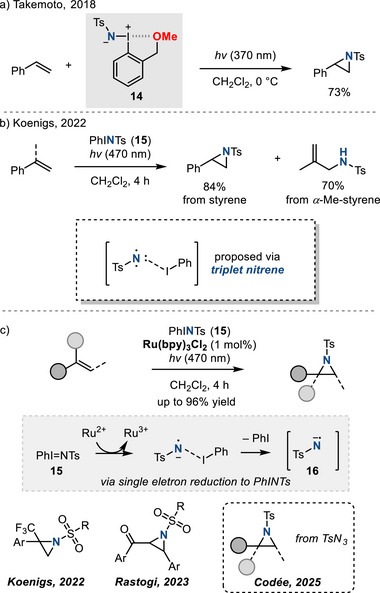
Photochemical aziridination with iminoiodinane reagents can proceed via photogenerated nitrenes or via nitrene radical anions.

Iminoiodinanes also represent useful nitrene photoprecursors for olefin aziridination chemistry. In 2018, Takemoto reported photochemical aziridination of styrene using *ortho*‐substituted iminoiodinane **14**.^[^ ^56^ ^]^ The introduction of a Lewis basic *ortho‐*CH_2_OMe group was essential to stabilize the photoexcited state of the iminoiodinane and prevent undesired nonselective reactions resulting from free nitrenes (Figure 13a). Takemoto's initial report described a single example of photochemical aziridination, by way of the synthesis of 2‐phenyl‐1‐tosylaziridine. Subsequently, Zhu extended the scope with >30 examples of *N*‐tosyl aziridines using blue LEDs and iminoiodinane **14**.^[^ ^57^ ^]^ This method is not stereospecific, and the authors proposed a stepwise mechanism via addition of a triplet nitrene intermediate to account for the observed mixture of *syn*/*anti* aziridines from *cis‐* and *trans*‐alkenes.

In 2022, Koenigs reported the photolysis of PhI = NTs (**15**) in the presence of styrenyl olefins affords the corresponding aziridination products (Figure 13b).^[^ ^58^ ^]^ In addition to olefin aziridination, products of competitive allylic amination were also observed for substrates with allylic C─H bonds such as α‐methyl‐styrene. The observed reactivity pattern was ascribed to the intermediacy of triplet nitrenes. Computational studies suggested that iminoiodinane photolysis initially results in a singlet excited state, which undergoes intersystem crossing and I─N bond cleavage to evolve iodobenzene and the triplet nitrene fragment. The triplet nitrene then adds to the olefinic substrate to initiate the observed nitrogen transfer chemistry. To avoid competitive allylic amination, the same authors introduced Ru(bpy)_3_Cl_2_ as a photocatalyst. Under these conditions, the corresponding nitrene radical anion **16** (i.e., formal one‐electron reduction of the triplet nitrene) is generated and selectively engages in olefin aziridination (Figure 13c).^[^ ^58^ ^]^ This key nitrene radical anion has been applied by the same group to synthesize trifluoromethylated aziridines.^[^ ^59^ ^]^ Rastogi subsequently extended the synthetic impact of nitrene radical anions to include aziridination of chalcone derivatives.^[^ ^60^ ^]^ In 2025, Codée et al. demonstrated that tosyl azide can also furnish nitrene radical anion reactivity to access aziridines from unactivated olefins.^[^ ^61^ ^]^

**Figure 14:**
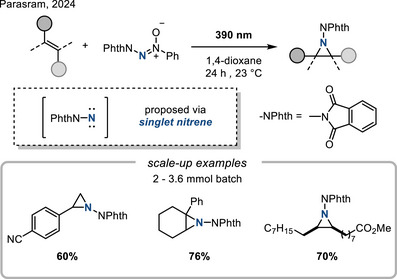
Photochemical synthesis of *N*‐phthalimidoaziridines from azoxy‐triazenes precursors.

In 2024, Parasram reported the synthesis of *N‐*phthalimidoaziridines from azoxy‐triazenes precursors under visible light irradiation (Figure 14).^[^ ^62^ ^]^ Mechanistic studies suggested the intermediacy of a singlet nitrene and concerted addition of the nitrogen group to olefins.^[^ ^63^ ^]^

**Figure 15:**
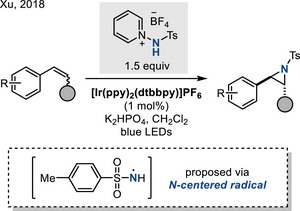
Photocatalytic aziridination with N─Ts aminopyridinium ylide.

Finally, *N*‐centered radicals derived from *N*‐aminopyridinium salts promote efficient aziridination of styrenes. This reaction is proposed to proceed via visible‐light‐mediated single‐electron reduction of *N*‐aminopyridinium salts by photoexcited Ir complex (Figure 15).^[^ ^64^ ^]^

#### 2.1.4 Electrochemical Aziridination

**Figure 16:**
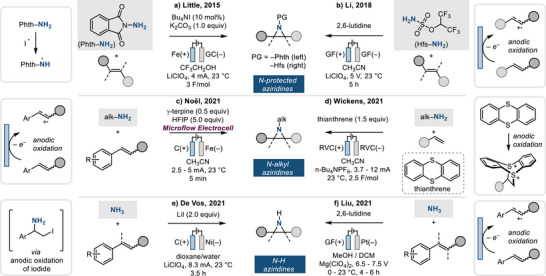
Electrochemical olefin aziridination to form *N*‐protected aziridines a) and b), *N*‐alkyl aziridines c) and d), and N─H aziridines e) and f).

Electrochemical olefin aziridination has recently emerged as complementary, and potentially sustainable, strategy for olefin aziridination. In the early 2000s, Yudin made seminal contributions to electrochemical olefin aziridination using *N*‐aminophthalimide as the nitrogen source.^[^ ^65^, ^66^ ^]^ Following these reports, Little developed a [TBA]I‐catalyzed aziridination method under constant current electrolysis ([TBA] = *tetra‐*butylammonium) (Figure 16a).^[^ ^67^ ^]^ In this reaction, electrochemically generated iodine atoms (i.e., I•) were proposed to mediate the generation of *N*‐centered radicals (NCRs), which then added to the olefinic partner to effect aziridination. In a complementary approach, Cheng disclosed electrochemical aziridination of triaryl‐substituted olefins (Figure 16b) by initial electrochemical olefin oxidation and subsequent trapping of the radical cation by sulfamates (i.e., Hfs‐NH_2_).^[^ ^68^ ^]^

Alkyl amines have also been utilized as the nitrogen source in electrochemical aziridination protocols. In 2021, Noël reported the electrochemical aziridination of internal olefins with alkyl amines to yield *N‐*alkyl aziridines (Figure 16c).^[^ ^69^ ^]^ This protocol proceeds through an electrochemically generated radical cation that is then trapped by the alkyl amine. As this method proceeds via a carbocation intermediate generated from olefin oxidation, unactivated terminal olefins are inefficient substrates. In 2021, Wickens developed olefin aziridination with alkyl amines via electrochemically generated dicationic thianthrenium intermediates (Figure 16d).^[^ ^70^ ^]^ Anodic oxidation of thianthrene initiates the olefin activation forming dicationic thianthrenium‐olefin adduct. This reaction works for unactivated terminal olefins probably due to steric congestion from dual involvement of thianthrenes. Both the Noël and Wickens strategies avoid amine prefunctionalization and are thus compatible with naturally occurring amine partners.

Electrochemistry has also enabled the use of ammonia in the synthesis of N─H aziridines. De Vos^[^ ^71^ ^]^ and Cheng^[^ ^72^ ^]^ reported the electrochemical aziridination of styrenyl olefins using ammonia to produce N─H aziridines under constant current (Figure 16e) and constant potential electrolysis (Figure 16f), respectively.^[^ ^71^, ^72^ ^]^ The latter method relies on initial anodic oxidation of the olefinic substrate and is thus limited to styrenyl olefins (i.e., olefins that generate stabilized benzylic radical cation intermediates).

### 2.2 Imine Aziridination (C1 + CN)

**Figure 17:**
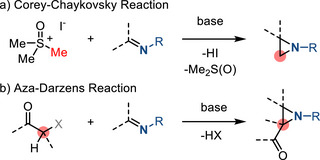
Aziridination of imines via a) Corey–Chaykovsky reaction and b) aza‐Darzens reaction.

In addition to the cycloaddition reactions between olefins and nitrene equivalents described above, aziridine rings can also be forged by the cycloaddition of imines with carbene equivalents. These methods are conceptually complementary to nitrene addition chemistry, and because carbene addition reactions do not rely on electrophilic nitrene equivalents, these methods can provide access to distinct *N*‐functionalization patterns. Historically, carbene‐based aziridine synthesis was demonstrated in the Corey–Chaykovsky reaction^[^ ^73^ ^]^ and aza‐Darzens reactions (Figure 17).^[^ ^74^ ^]^

**Figure 18:**
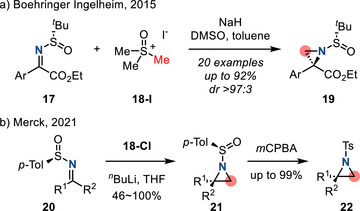
Diastereoselective Corey–Chaykovsky reaction of chiral *N*‐sulfinyl imines to form aziridines.

Recent progress in Corey–Chaykovsky reaction has focused on the development of stereoselective aziridine syntheses. For example, Marsini et al. (Boehringer Ingelheim) applied the Corey–Chaykovsky reaction to chiral *N*‐*tert*‐butanesulfinyl ketimino esters **17** to form *N*‐sulfinyl aziridines **19** in good diastereoselectivity (Figure 18a).^[^ ^75^ ^]^ Apgar et al. (Merck) used a related strategy in the synthesis of Ibrexafungerp, an orally active β‐1,3‐glucan synthase inhibitor. The Merck synthesis proceeded through (*R*)‐α‐disubstituted *N*‐tosylaziridine **22**. Attempts to access this intermediate through olefin aziridination resulted in racemic diastereomeric mixtures. In comparison, Corey–Chaykovsky reaction with chiral sulfinyl imine **20** provided access to enantiopure aziridine **21**; subsequent oxidation furnished *N*‐tosylaziridine **22** (Figure 18b).^[^ ^76^ ^]^ Examples of Corey–Chaykovsky reaction via ammonium ylides have also been recently reported.^[^ ^77^, ^78^, ^79^ ^]^

**Figure 19:**
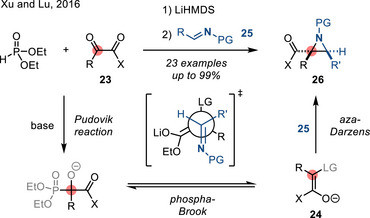
Diastereoselective aza‐Darzens reaction to form *N*‐protected 2‐carbonyl aziridines (LG = OP(O)(OEt)_2_, PG = P(O)(OPh)_2_).

Similarly, recent progress in aza‐Darzens reaction has focused on stereoselective methods. In 2016, Xu and Lu coupled imines with α‐ketoesters to access aziridine‐2‐carboxylates (Figure 19).^[^ ^80^ ^]^ In this reaction, α‐ketoester **23** underwent Pudovik reaction with diethyl phosphite. Subsequent phospha‐Brook rearrangement afforded enolate **24** which then added to the imine partner **25** to afford aziridines **26**. The authors reported good diastereoselectivities (dr > 20:1) and high yields for *N*‐diphenylphosphinyl imines.

**Figure 20:**
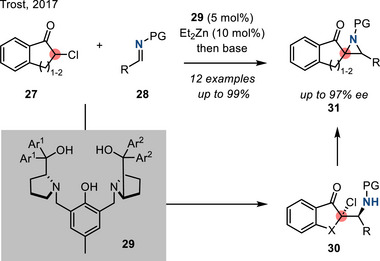
Enantioselective aza‐Darzens reaction to form chiral trisubstituted aziridines via Lewis acid catalysis (PG = Boc or Cbz, Ar^1^ = 4‐(CF_3_)C_4_H_6_, Ar^2^ = 4‐(MeO)C_4_H_6_).

In 2017, Trost reported a catalytic enantioselective aza‐Darzens reaction between cyclic α‐chloroketones and *N‐*carboxy imines to access chiral nonracemic trisubstituted aziridines.^[^ ^81^ ^]^ In this protocol, an enantioselective Mannich reaction between **27** and **28** was promoted by chiral Zn‐ProPhenol catalyst **29** to generate a 1,2‐chloroamine intermediate. Upon treatment with base, **30** cyclized to the corresponding aziridines **31** (Figure 20). *N*‐Boc and *N*‐Cbz imines were competent substrates in this scheme.

**Figure 21:**
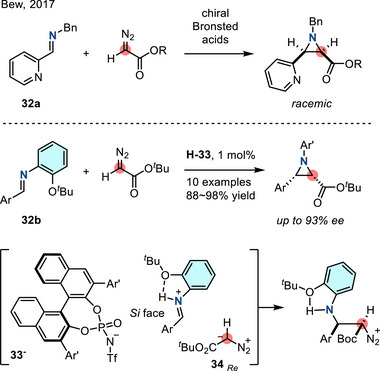
Enantioselective aza‐Darzens reaction via chiral Bronsted acid catalysis (Ar’ = 2‐(*^t^* BuO)C_6_H_4_).

Chiral Bronsted acid‐catalyzed aziridination of α‐diazoesters and imines has attracted attention as a strategy to access enantioenriched disubstituted aziridines.^[^ ^82^, ^83^ ^]^ In 2017, Bew reported an enantioselective aza‐Darzens reaction with azoacetates to afford chiral *N*‐aryl aziridines (Figure 21).^[^ ^84^ ^]^ In this report, the authors demonstrated the impact of the imine *N*‐substituent (i.e., **32**) on the reaction enantioselectivity: *N*‐benzyl imines (**32a**) underwent aza‐Darzens reaction to form racemic aziridines, while *N‐ortho*‐*tert*‐butylphenyl imines (**32b**) engaged in enantioselective aziridination. This dichotomous outcome was attributed to intramolecular hydrogen bonding of the protonated imine, restricting the conformation during the enantioselective nucleophilic addition with **34_Re_**.

**Figure 22:**
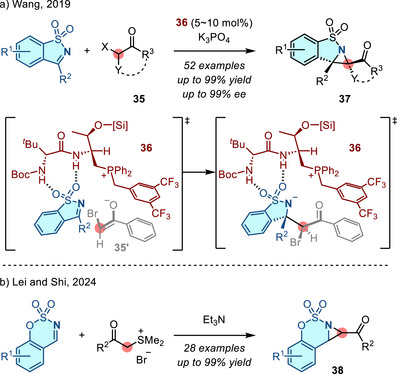
Aziridination of cyclic sulfonyl imines via a) chiral phosphonium catalyzed asymmetric aza‐Darzens reaction and b) Corey–Chaykovsky reaction.

Cyclic *N*‐sulfonyl imines participate in both aza‐Darzens and Corey–Chaykovsky reactions. In 2019, Wang reported the enantioselective synthesis of fused aziridines **37** from cyclic sulfonyl imines and α‐halogenated ketones **35** under the action of chiral phosphonium catalyst **36**.^[^ ^85^ ^]^ Enantioselectivity was proposed to arise from hydrogen bonding between the catalyst and the sulfonyl group of the substrate (Figure 22a). Similarly, in 2024, Lei and Shi applied the Corey–Chaykovsky reaction to access fused aziridines **38** with sulfonium salts (Figure 22b).^[^ ^86^ ^]^

**Figure 23:**
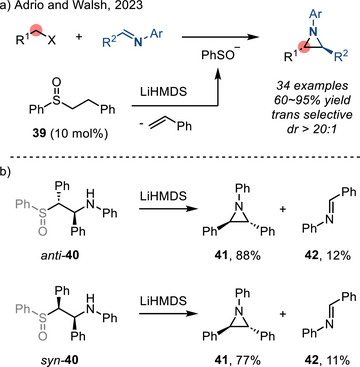
a) Diastereoselective imine aziridination catalyzed by in situ generated sulfenate anion. b) Control experiments accounted for the reaction diastereoselectivity.

In 2023, Adrio and Walsh described an organocatalytic diastereoselective aziridination reaction of *N*‐aryl imines with sulfur ylides. In this reaction, the ylide was generated by nucleophilic addition of sulfenate (PhSO^−^), generated by base‐promoted elimination of **39**, to alkyl halides (Figure 23a).^[^ ^87^ ^]^ This method provided access to 1,2‐disubstituted *N*‐aryl aziridines with high *trans*‐diastereoselectivity, which was a marked improvement over previous methods and attempts with other commonly encountered *N*‐functionalities.^[^ ^88^ ^]^ Control experiments using *syn*‐ and *anti*‐**40** resulted in the formation of *trans*‐aziridine **41** along with the imine **42** (Figure 23b). These observations indicate that ylide addition to the imine was reversible and that potential ring closure of *syn*‐**40** was not productive. *Anti*‐**40** underwent rapid ring closure to favor formation of *trans*‐aziridine **41**.

**Figure 24:**
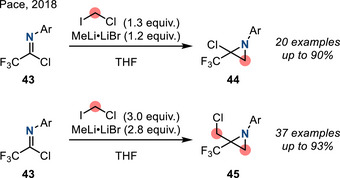
Sequential homologation of imidoyl chlorides with carbenoids to access aziridines.

*N‐*Aryl imidoyl chlorides engage with carbene equivalents to afford aziridine homologation products. In 2018, Pace reported the combination of trifluoroacetimidoyl chlorides **43** with lithium carbenoids (LiCH_2_X) (Figure 24): Homologation of imines **43** formed 2‐chloroaziridines **44** using 1 equivalent of LiCH_2_X, while sequential homologation gave 2‐chloromethyl aziridines **45** using a second equivalent of LiCH_2_X.^[^ ^89^ ^]^

### 2.3 Intramolecular Cyclization

While carbene and nitrene transfer reactions dominate the synthetic chemistry of aziridines, these reactions are not endemic to the biosynthesis of aziridines. In lieu of cycloaddition chemistry, aziridine biosynthesis typically proceeds via intramolecular cyclization of β‐functionalized amine derivatives.^[^ ^90^ ^]^ Inspired by these intramolecular cyclization reactions, the past 10 years have witnessed the development of a family of intramolecular cyclization strategies via both chemical and biochemical pathways. In this section, we first discuss modern methods to prepare aziridines via cyclization of β‐functionalized amine derivatives. Then, we will discuss emerging methods in aziridine synthesis via β‐C─H activation of aliphatic amines. For both, chemical and biocatalytic aziridinations will be discussed.

#### 2.3.1 Intramolecular Nucleophilic Substitution

**Figure 25:**
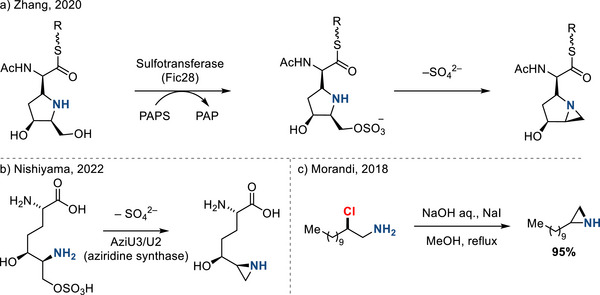
Biocatalytic cyclization of 1,2‐aminosulfates to access aziridines (a and b), and c) cyclization of 2‐chloroalkylamines for aziridine synthesis.

Aziridine biosynthesis typically proceeds via intramolecular cyclization of β‐functionalized amine derivatives.^[^ ^2^ ^]^ 3‐*Exo*‐tet cyclization of these substrates is typically facile, resulting in efficient ring‐closing reactions. For example, Zhang demonstrated that sulfotransferase enzymes generate aziridines via cyclization of amine precursors following sulfation of proximal hydroxyl groups (Figure 25a).^[^ ^91^ ^]^ Similar sulfate‐displacement pathways have been implicated by Nishiyama in AziU3/U2, which is functionally an aziridine synthase (Figure 25b).^[^ ^92^ ^]^

#### 2.3.2 From Allyl Amines

Cyclization of 2‐functionalized (e.g., sulfonate) amines is among the earliest methods to construct aziridines, known as the Wenker synthesis.^[^ ^93^ ^]^ Contemporary efforts often couple this strategy with olefin 1,2‐aminofunctionalization: For example, in 2018, Morandi demonstrated cyclization of 2‐chloroalkylamines to afford N─H aziridines (Figure 25c).^[^ ^94^ ^]^ More recently, the same intramolecular cyclization logic has been implemented in the context of aziridine synthesis from allyl amines, which is advantageous because the allyl group can be carried through multistep synthetic sequences and aziridine formation proceeds only when the π‐bond is purposefully activated. Broadly, two strategies have been adopted: a) Hypervalent iodine(III)‐mediated olefin aminofunctionalization and b) radical‐initiated olefin aminofunctionalization.

**Figure 26:**
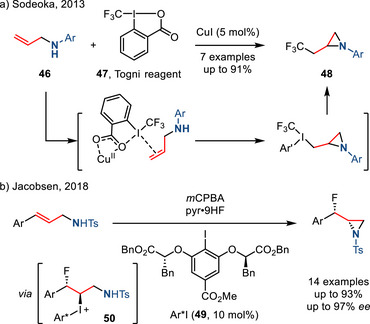
Hypervalent iodine(III)‐mediated aminofunctionalization of allylamines to access a) trifluoromethylated and b) fluorinated aziridines.

*Iodine(III)‐Mediated Olefin Aminofunctionalization*. In 2013, Sodeoka reported the copper‐catalyzed aminotrifluoromethylation of allylamines **46** using Togni reagent (**47**) to afford aziridines **48**.^[^ ^95^ ^]^ In 2015, the same group reported an investigation of the mechanism of this reaction. They proposed that Cu(II), generated by in situ oxidation of the Cu(I) precatalyst, serves as a Lewis acid to enhance the electrophilicity of the iodine(III) center (Figure 26a).^[^ ^96^ ^]^ Owing to the importance of vicinal fluoroamine substructure in medicinal chemistry,^[^ ^97^, ^98^, ^99^, ^100^ ^]^ Jacobsen developed the I(III)‐mediated fluoroamination of allylamines.^[^ ^101^ ^]^ This reaction utilized pyridine‐HF as the fluoride source, *m*CPBA as the terminal oxidant, and C2‐symmetric aryl iodide **49** as the redox catalyst. Formation of the aziridine ring was proposed to proceed by intramolecular nucleophilic displacement of an aryl iodide leaving group in iodonium intermediate **50** (Figure 26b).

**Figure 27:**
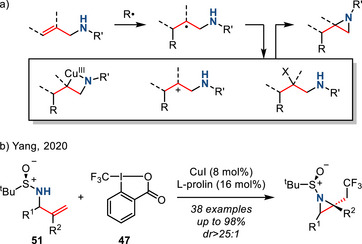
a) General scheme and intermediates of radical‐initiated aziridine synthesis from allylamines; b) Cu‐catalyzed diastereoselective aziridine synthesis via trifluoromethyl radical.

*Radical‐Initiated Olefin Aminofunctionalization*. Allyl amines are also precursors to aziridines via radical‐mediated cyclizations (Figure 27a). For example, addition of carbon‐centered radicals to allylamines can generate β‐amino radicals. These radicals can either chain‐propagate via halogen abstraction from starting alkyl halides or undergo radical polar crossover to form alkyl halides or carbocations. Subsequent ring closure gives access to a wide range of β‐substituted aziridines. For example, Yang reported the diastereoselective aminotrifluoromethylation of *N*‐sulfinyl allylamines **51** with Togni's reagent in the presence of copper catalyst (Figure 27b). In contrast to Sodeoka's report (Figure 26a),^[^ ^96^ ^]^ the authors proposed a mechanism involving trifluoromethyl radical addition.^[^ ^102^ ^]^

**Figure 28:**
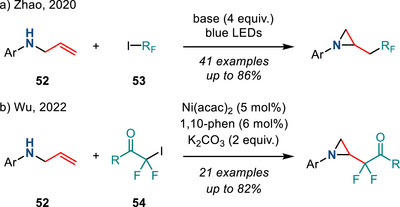
a) Photochemical and b) Ni‐catalyzed methods to access fluoroalkylated *N*‐aryl aziridines from *N*‐allylanilines.

In 2020, Zhao reported a catalyst‐free photochemical aziridine synthesis from *N*‐allylanilines and fluoroalkyl iodides (Figure 28a).^[^ ^103^ ^]^ This reaction was proposed to proceed via a halogen‐bonded intermediate assembled by interaction of amine **52** and the C─I bond in **53**. Fluoroalkyl radicals were subsequently generated upon visible light irradiation. In 2022, Wu disclosed a related transformation in which a fluoroalkyl radical, generated from iododifluoromethyl ketones (**54**) and a Ni catalyst, initiated cyclization (Figure 28b).^[^ ^104^ ^]^

**Figure 29:**
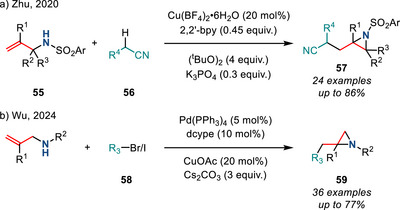
a) Cu‐ and b) Pd‐catalyzed aminoalkylation of allyamines to form 2‐alkyl aziridines.

In 2020, Zhu demonstrated that alkyl nitriles are also viable radical precursors in the Cu‐catalyzed aziridination of *N*‐sulfonyl allylamines **55** to form aziridines **57** (Figure 29a).^[^ ^105^ ^]^ In this reaction, H‐atom abstraction from nitrile **56** using di‐*tert*‐butylperoxide generated the corresponding cyanoalkyl radical, which added to **55** to initiate aziridination. Pd‐catalyzed radical generation has also been investigated to efficiently construct aziridines **59** from alkyl halides **58**, as described by Wu in 2024 (Figure 29b).^[^ ^106^ ^]^

**Figure 30:**
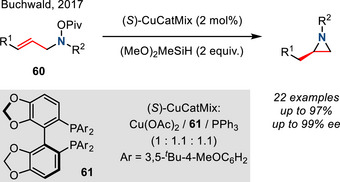
Cu‐catalyzed intramolecular hydroamination of *N*‐OPiv allyl amines to access aziridines.

Finally, Buchwald demonstrated an intramolecular enantioselective aziridination of *N*‐OPiv‐substituted allylic amines **60** via Cu hydride catalysis (Figure 30). In this reaction, regioselective hydrocupration of the allyl amine generated the organocopper intermediate, which subsequently underwent intramolecular amination to furnish chiral aziridines. This cycloamination methodology accessed nonactivated alkyl‐substituted enantioenriched aziridines in high yields, high regio‐ and stereocontrol. However, N─O reduction was predominant when using (*Z*)‐alkenes as the substrate.^[^ ^107^ ^]^

#### 2.3.3 β‐C─H Activation of Amines

In concept, β‐C─H activation of amines is a potential aziridine disconnection that avoids oxidative substrate prefunctionalization. From a synthetic perspective, this connection is relatively underdeveloped but holds significant promise for rapid elaboration of nitrogen‐containing small molecules and opportunities to rapidly access aziridines.

**Figure 31:**
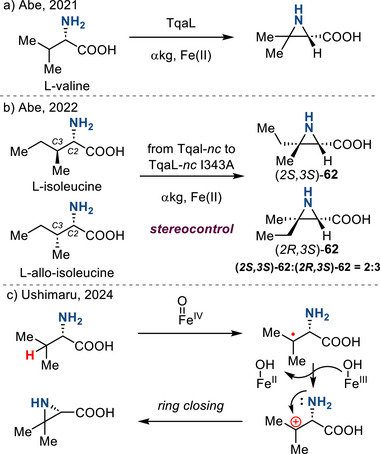
a) Enzymatic access to aziridines from l‐valine. b) Stereoselective access to aziridines via protein engineering. c) Mechanism of Fe‐catalyzed enzymatic aziridine synthesis via β‐C─H activation.

Enzymatic β‐C─H activation of amines can provide access to aziridines. In 2021, Abe reported a previously unknown aziridination pathway from l‐valine with the nonheme iron and α‐ketoglutarate‐dependent (FeII/αKG) oxygenase TqaL (Figure 31a).^[^ ^108^ ^]^ Using either l‐isoleucine or l‐allo‐isoleucine as starting materials afforded the same 2:3 ratio of (2*S*,3*S*)‐**62** and (2*R*,3*S*)‐**62**. The lack of stereospecificity suggested the intermediacy of carbon‐centered radicals (or cations), which are susceptible to C2–C3 rotation, as intermediates in this enzyme‐promoted aziridination. Systematic analysis by crystal structure prediction by AlphaFold2, followed by mutation on TqaL from *Neurospora crassa* (TqaL‐*nc*) at Ile343 and Phe345 positions, resulted in an enzymatic catalyst that promoted aziridination with 1:3 *dr*, which demonstrated the opportunity of rational control of stereoselectivity by protein engineering (Figure 31b).^[^ ^109^ ^]^

Chang further elucidated the mechanism of this enzyme‐mediated aziridination and developed a TqaL‐*ha*, a variant of TqaL, which selectively affords (2*R*,3*S*)‐**62** as a single diastereomer.^[^ ^110^ ^]^ Further mutation of TqaL afforded a pair of variants, TqaL‐ti and TqaL‐*ti* I295, that selectively afford two different diastereomers (2*S*,3*S*)‐**62** and (2*R*,3*S*)‐**62**.^[^ ^111^ ^]^ Mechanistic findings suggest that an Fe(IV)‐oxo effects β‐C─H abstraction to generate an Fe(III)─OH and a β‐amino radical. Subsequent single‐electron oxidation of the β‐amino radical by the Fe(III)─OH affords a β‐amino carbocation, which undergoes cyclization to the observed aziridines (Figure 31c).^[^ ^112^ ^]^

**Figure 32:**
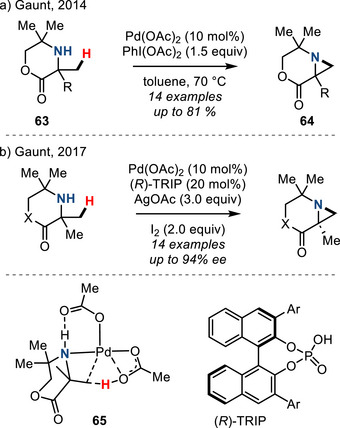
Pd‐catalyzed oxidative intramolecular N─H/C─H coupling to access aziridines.

In 2014, Gaunt described a chemocatalytic method for aziridination via β‐C─H activation. In this method, a Pd catalyst promotes intramolecular oxidative C─H/N─H coupling of the morpholin‐2‐one substrates **63** to afford aziridines **64** in good yields (Figure 32a). This was achieved by the C–H palladation of the methyl group to form a 4‐membered aza‐palladacycle followed by reductive elimination to form the C─N bond.^[^ ^113^ ^]^ Further mechanistic investigations revealed that H‐bonding‐controlled concerted metalation−deprotonation pathway (**65** in Figure 32) plays a key role in C−H activation.^[^ ^114^ ^]^ DFT studies suggested that CMD (concerted metalation–deprotonation) with the methyl group nearest the carbonyl is lower in energy than that at the methyl groups furthest from the carbonyl, which accounts for the observed regioselectivity. In 2017, Gaunt demonstrated an enantioselective version of this reaction by employing chiral anionic BINOL‐phosphoric acid ligands (Figure 32b).^[^ ^115^ ^]^ While this is a conceptually attractive strategy and a rare example of aziridine synthesis via β‐C─H activation, at present the substrate scope is limited to morpholin‐2‐one and piperazin‐2‐one scaffolds.

### 2.4 Miscellaneous Methods

A number of aziridine‐forming reactions have been developed that are mechanistically distinct from the broad categories discussed above. Below, we discuss aziridine syntheses based on functionalization of 2H‐azirine precursors and based on Baldwin rearrangement of 4‐isoxazolines.

#### 2.4.1 Addition/Insertion to 2*H*‐Azirines

**Figure 33:**
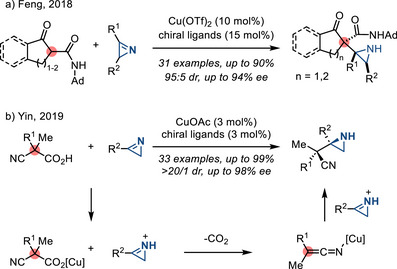
Enantioselective access to N─H aziridines from 2*H*‐azirines via a) chiral Cu(II) catalysis with β‐ketoamides, and b) chiral Cu(I) catalyzed decarboxylative alkylation.

In 2018, Feng reported the asymmetric synthesis of N─H aziridines via the addition of tertiary carbon nucleophiles (from β‐ketoamides) to 2*H*‐azirines in the presence of a chiral Cu(II) catalyst (Figure 33a).^[^ ^116^ ^]^ In 2019, Yin demonstrated aziridine synthesis via addition of nitrile enolates, generated by decarboxylation of acid precursors, to 2*H*‐azirines under the action of a chiral Cu(I) catalyst.^[^ ^117^ ^]^ where the electrophilicity was enhanced by protonation of the 2*H*‐azirine substrate (Figure 33b).

**Figure 34:**
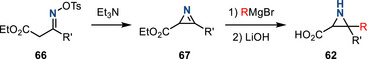
Synthesis of aziridines **62** via sequential intramolecular substitution of *O*‐tosyl oximes to afford 2*H‐*azirines followed by nucleophilic addition with Grignard reagents (R,R’ = Me, Et).

Grignard reagents add to 2*H*‐azirines to afford aziridines (Figure 34). For example, aziridines **62** were synthesized via nucleophilic addition of alkyl Grignard reagents to 2*H*‐azirines **67**. These aziridines were used to probe stereoselectivity and substrate specificity of the enzyme‐catalyzed aziridination depicted in Figure 31. Azirine precursors **67** were accessed by intramolecular substitution of *O*‐tosyl oxime **66**.^[^ ^109^ ^]^

**Figure 35:**
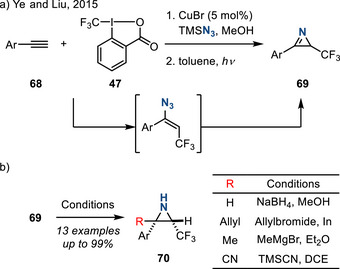
a) Cu(I)‐catalyzed alkyne trifluoromethylative azidation to form *2H‐*azirines. b) Nucleophilic addition to *2H‐*azirines provides access to N─H aziridines.

In 2015, Ye and Liu reported a two‐step sequence to prepare 2*H*‐azirines **69** comprised of 1) trifluoromethylative azidation of alkynes **68** followed by 2) photolysis of the resulting vinyl azides (Figure 35a).^[^ ^118^ ^]^ Carbon‐based nucleophiles (allylindiums, methyl magnesium halides, and cyanide salts), as well as hydride reagents, participate in the nucleophilic addition of the resulting azirines **69** to form N─H aziridines **70** (Figure 35b).

**Figure 36:**
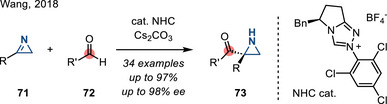
NHC‐catalyzed enantioselective aza‐benzoin reaction of 2*H*‐azirines to access chiral 2‐carbonyl aziridines.

2*H*‐Azirines also engage in the aza‐benzoin reaction to afford N─H aziridines. In 2018, Wang reported an enantioselective aza‐benzoin reaction between azirines **71** and aldehydes **72** promoted by a chiral NHC catalyst, affording chiral aziridines **73** in good yields with high enantioselectivities (Figure 36).^[^ ^119^ ^]^

**Figure 37:**
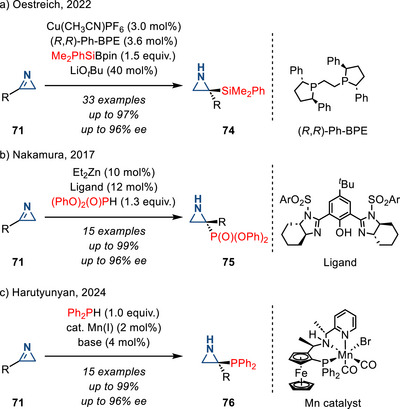
Enantioselective addition of silicon‐ and phosphorus‐based nucleophiles to 2*H*‐azirines provides entry to a) 2‐aziridinyl silanes, b) phosphonates, and c) phosphines (Ar = mesityl).

Heteroatom‐based nucleophiles also participate in enantioselective addition to 2*H*‐azirines: Using silyl pinacol borane nucleophiles, Oestreich reported a Cu(I)‐catalyzed enantioselective *C*‐silylation of 2*H*‐azirines (Figure 37a).^[^ ^120^ ^]^ The stereoselectivity of this reaction was proposed to arise from steric control of the nucleophile approach to a C2‐symmetric intermediate. In 2017, Nakamura reported a Zn(II)‐catalyzed enantioselective *C*‐phosphonation of 2*H*‐azirines with a chiral phenol‐bis(imidazoline) ligand (Figure 37b).^[^ ^121^ ^]^ Enantioinduction was proposed to arise from an intermediate in which Zn(II) coordinates to a nitrogen of the imidazoline and the oxygen of the chiral phenol. The diphenyl phosphite was proposed to engage in an H‐bond with the other imidazoline moiety, thus enforcing nucleophilic addition from the *Re*‐face of the azirine. In 2024, Harutyunyan reported a Mn(I)‐catalyzed enantioselective *C*‐phosphination of 2*H*‐azirines using diphenylphosphine (Figure 37c).^[^ ^122^ ^]^ This reaction was proposed to proceed via a Mn‐phosphido intermediate, where the N─H moiety of the ligand formed a hydrogen bond with **71**, followed by stereoselective addition of the phosphide ligand. In contrast to broadly studied olefin aziridination strategies, these methods provide 2‐heteroatom functionalized aziridines (**74**–**76**), expanding the chemical space in aziridine synthesis.

#### 2.4.2 Baldwin Rearrangement of 4‐Isoxazolines

**Figure 38:**
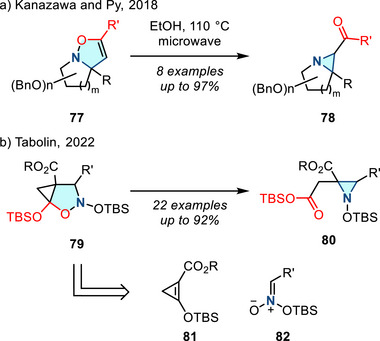
a) Baldwin rearrangement of cyclic 4‐isoxazolines to access 2‐acyl aziridines. b) Homo‐Baldwin rearrangement to form aziridines.

Lastly, Baldwin rearrangement, which is a thermally induced ring contraction of 4‐isoxazolines, can also be used to access aziridines.^[^ ^123^ ^]^ Since the discovery of this reaction in 1968, this aziridination method has not received significant attention. Recently there have been several advances in this synthetic space: In 2017, Kanazawa and Py reported Baldwin rearrangement of cyclic 4‐isoxazolines **77** to access 1‐azabicyclic aziridines **78** (Figure 38a), which served as iminosugar‐based ABPs (vide infra).^[^ ^124^ ^]^ In 2022, Tabolin reported a homo‐Baldwin rearrangement of an isoxazolidine system (Figure 38b). Aziridines **80** were formed from the intermediate **79**, which resulted from the 1,3‐dipolar cycloaddition between cyclopropene **81** and nitronate **82**.^[^ ^125^ ^]^ In 2023, flow process was introduced to the Baldwin rearrangement, extending the substrate scope (15 examples, up to 84% yield).^[^ ^126^ ^]^ Related photochemical rearrangements have also been reported for the construction of aziridines.^[^ ^127^, ^128^ ^]^

## 3 *N*‐(De)functionalization of Aziridines

The exocyclic *N*‐substituent of aziridines has a profound effect on the reactivity of the strained ring.^[^ ^1^ ^]^ Aziridines bearing electron‐withdrawing *N*‐substituents are generally more electrophilic and thus more susceptible to ring‐opening and ‐expansion chemistry; nucleophilic addition to these strained rings typically proceeds at the less‐hindered carbon. In contrast, aziridines bearing electron‐neutral or ‐donating *N*‐substituents tend to display suppressed electrophilicity and more varied regioselectivity in ring‐opening reactions.^[^ ^1^, ^129^ ^]^ As described above, most aziridine syntheses require specific *N‐*functional group for efficient aziridination reactions; for example, most metal‐catalyzed nitrene reactions require electron‐withdrawing *N*‐substituents to engender efficient aziridine construction. Thus, removal or derivatization of these substituents is necessary to access the potential chemical space of aziridine‐containing small molecules. Given the proclivity of aziridines to engage in ring‐opening reactions, adaptation of the reaction chemistry of acyclic amines to aziridines is not straightforward and often specific conditions must be developed in the context of aziridine chemistry.

### 3.1 *N‐*Deprotection to Form N─H Aziridines

#### 3.1.1 Removal of *N*‐sulfonyl(sulfinyl) Substituents

**Figure 39:**
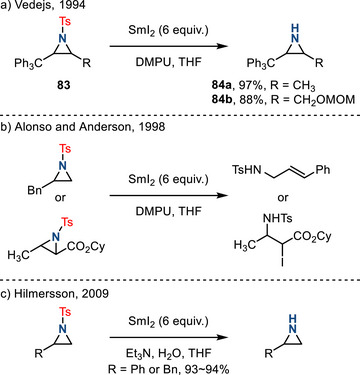
Reductive conditions for the removal of N─Ts group: a) N─Ts cleavage promoted by SmI_2_; b) ring‐opening of N─Ts aziridines under SmI_2_ conditions; c) Et_3_N‐mediated desulfonylation of aziridines with SmI_2_.

*N*‐Sulfonyl aziridines are among the most often prepared derivatives due to the ubiquity of *N*‐sulfonyl nitrene transfer in metal‐catalyzed aziridination chemistry.^[^ ^17^ ^]^ *N*‐Sulfonyl substituents are typically removed under reducing conditions. In 1994, Vedejs reported the deprotection of arenesulfonamides **83** using SmI_2_ with DMPU in refluxing THF (DMPU = *N*,*N’*‐dimethylpropyleneurea).^[^ ^130^ ^]^ The substrate scope in this initial report was limited to 2 examples, **84a** and **84b** (Figure 39a). In 1998, Alonso and Anderson reported attempts to remove *N*‐sulfonyl substituents under similar conditions but obtained products of ring‐opening chemistry, not aziridine deprotection (Figure 39b).^[^ ^131^ ^]^ In 2009, Hilmersson reported the combination of SmI_2_, a tertiary amine, and water effected efficient removal of N─Ts substituents to afford the corresponding secondary amines (Figure 39c).^[^ ^132^ ^]^ These conditions were compatible with *N*‐tosyl aziridine deprotection, albeit only two examples (*N*‐tosyl‐2‐phenylaziridine and *N*‐tosyl‐2‐benzylaziridine) were demonstrated. In these conditions, the identity of the tertiary amine additive was decisive: Pyrrolidine or isopropylamine gave no isolated product, while triethylamine afforded the desired N─H aziridine in excellent yields.

**Figure 40:**
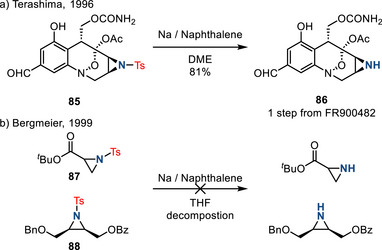
a) Treatment of N─Ts aziridine **85** with Na/naphthalene effected desulfonylation during the synthesis of FR900482. b) Substrates incompatible with Na/naphthalene conditions.

Birch‐type conditions have been applied to removal of *N*‐sulfonyl groups from aziridines (Figure 40). In 1996, Terashima reported the conversion of N─Ts aziridine **85** to N─H aziridine **86** by treatment with sodium naphthalenide during the total synthesis of FR‐900482 (Figure 40a).^[^ ^133^ ^]^ In 1999, Bergmeier demonstrated that these conditions represent a general approach to N─Ts cleavage:^[^ ^134^ ^]^ Eight examples were demonstrated in 64%–93% yields. Reductively labile substrates, such as **87** and **88**, were incompatible with the developed conditions and afforded decomposition (Figure 40b).

**Figure 41:**
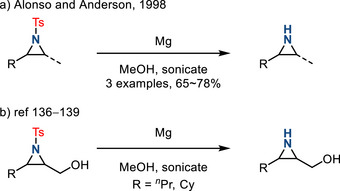
a) Early reports of Mg‐promoted N─Ts cleavage. b) More recent examples of Mg‐promoted synthesis of N─H aziridines.

In 1998, Alonso and Anderson reported the removal of *N*‐sulfonyl groups by sonication in the presence of magnesium in methanol (Figure 41a).^[^ ^131^ ^]^ These conditions resulted in less undesired ring‐opening chemistry than the SmI_2_‐promoted N─S cleavage. In 2004, Chang introduced 2‐pyridinesulfonyl aziridines via a Cu‐catalyzed olefin aziridination reaction.^[^ ^135^ ^]^ The resulting sulfonyl aziridines underwent more efficient and selective deprotection compared to the toysl aziridines upon treatment with magnesium. Mg‐based cleavage of N─Ts groups continues to be applied and has developed as a fairly versatile synthetic condition (Figure 41b).^[^ ^136^, ^137^, ^138^, ^139^ ^]^

**Figure 42:**
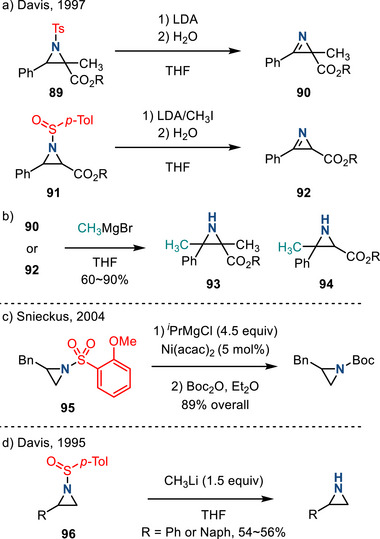
a) Access to 2*H*‐azirines from N─Ts or *N*‐sulfinyl aziridines by treatment with LDA. b) Nucleophilic addition to 2*H*‐azirines to form N─H aziridines. c) Desulfonylation of *o*‐anisylsulfonyl group by treatment with *^i^* PrMgCl and catalytic Ni(acac)_2_. d) Removal of sulfinyl group with methyl lithium to form N─H aziridines.

Organometallic reagents and strong bases have also been used to promote the removal of *N‐*sulfonyl and *N‐*sulfinyl aziridine substituents: In 1997, Davis reported LDA‐promoted elimination from N─Ts aziridines **89** to form 2*H*‐azirines **90** (Figure 42a). Subsequent nucleophilic addition of MeMgBr to the C═N bond afforded 2‐methyl aziridines **93** (Figure 42b).^[^ ^140^ ^]^ The *ortho*‐anisylsulfonyl group has also been employed in aziridine chemistry. In 2004, Snieckus demonstrated Ni‐catalyzed deprotection of *o*‐anisylsulfonamides with *^i^* PrMgCl.^[^ ^141^ ^]^ This method tolerated 5‐ and 6‐membered *N*‐heterocycles as well as acyclic sulfonamides, affording secondary amines. Removal of the *o*‐anisylsulfonyl group from aziridines **95** can be achieved by treatment with *^i^* PrMgCl in the presence of a Ni catalyst (Figure 42c). This report focused on generic deprotection of *o*‐anisylsulfonamides, and as a result, the scope of aziridine deprotection was not extensively investigated.

*N*‐Sulfinyl substituents can also be removed with similar strategies: In 1995, Davis reported the diastereoselective synthesis of *N*‐sulfinyl aziridines **96** via a Darzens‐type addition to an enantiopure sulfinimine.^[^ ^142^ ^]^ Treatment with MeLi afforded the N─H aziridines (Figure 42d).^[^ ^142^ ^]^ In 1997, the same authors treated *N*‐sulfinyl aziridines **91** with LDA/MeI to form 2*H*‐azirines **92**; subsequent MeMgBr addition resulted in 2‐methyl aziridines **94** (Figure 42b).^[^ ^140^ ^]^

**Figure 43:**
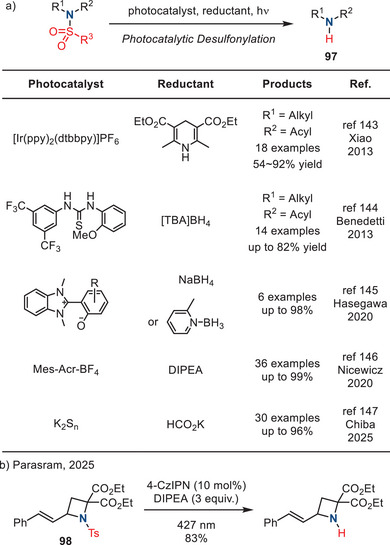
a) Photocatalytic removal of *N‐*sulfonyl groups to form secondary amines. b) Photocatalytic deprotection of N─Ts azetidines.

Recently, reports of photocatalytic deprotection of sulfonamides have emerged.^[^ ^143^, ^144^, ^145^, ^146^, ^147^ ^]^ These methods provide access to secondary amides, anilines, and amines (**97**), which are summarized in Figure 43a. Desulfonylation of azetidines (**98**) has also been reported by Parasram (Figure 43b).^[^ ^148^ ^]^ Desulfonylation of N─Ts aziridines, however, has not been fully explored yet.

**Figure 44:**
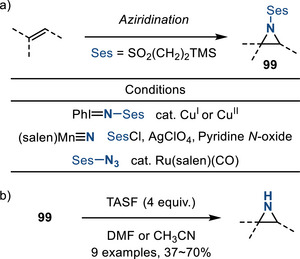
a) Synthesis of N─Ses aziridines can be accomplished with iminoiodinanes, manganese nitrides, and azides; b) N─Ses cleavage using fluoride to form N─H aziridines.

*N‐*Trimethylsilylethansulfonyl (N─Ses) groups have found application in aziridine chemistry and are attractive because they can often be removed via a fluoride‐promoted process that does not require strongly reducing conditions.^[^ ^149^ ^]^ In 1999, Dauban and Dodd introduced trimethylsilylethansulfonyl group as the activating group for aziridination (via Cu‐catalyzed nitrogen group transfer with PhI = NSes).^[^ ^150^ ^]^ They demonstrate cleavage of the N─Ses group by treatment with TASF (tris(dimethylamino)sulfonium difluorotrimethylsilicate). In 2002, Komatsu reported SesCl as a terminal electrophile in the nitrogen‐atom transfer chemistry with stoichiometric manganese nitride, forming N─Ses aziridines **99** (Figure 44a).^[^ ^151^ ^]^ Later, Katsuki utilized the azide reagent (SesN_3_) for metal‐catalyzed nitrene group transfer, forming aziridines in improved yields.^[^ ^152^, ^153^ ^]^ Overall, nine examples of TASF‐promoted N─Ses cleavage were described (Figure 44b). Notably, the α‐protons of N─Ses amines are sufficiently acidic to be abstracted by strong bases, while this is not the case for N─Ts amines.^[^ ^149^ ^]^

#### 3.1.2 Removal of *N*‐Acyl Substituents

**Figure 45:**
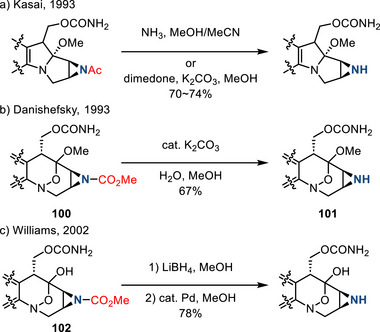
Removal of *N*‐acyl or carboxylate groups in natural product synthesis by a) solvolysis, b) base‐catalyzed hydrolysis, and c) reduction.

*N‐*Carboxyl and *N‐*acyl groups are common *N*‐activating groups for olefin aziridination chemistry.^[^ ^154^ ^]^ Historically, solvolysis was used to achieve deprotection: In 1993, Kasai reported the removal of an *N*‐acyl group in the presence of ammonia during the synthesis of 6‐demethyl mitomycin C (Figure 45a).^[^ ^155^ ^]^ In the same year, the *N*‐carboxyl aziridine **100** was hydrolyzed in MeOH with catalytic base (K_2_CO_3_) to form N─H aziridine **101** during the synthesis of congeners of FR‐900482 by Danishefsky (Figure 45b).^[^ ^156^ ^]^ *N*‐Carboxyl aziridine **102** could also be reduced by LiBH_4_, followed by Pd‐catalyzed decomposition of the resulting borane–aziridine complex (Figure 45c), which was applied to the synthesis of FR‐66979 and FR‐900482.^[^ ^157^ ^]^

**Figure 46:**
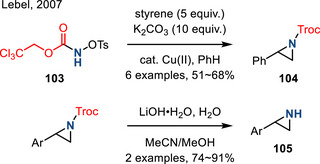
Styrene aziridination with TsO─NHTroc, followed by hydrolysis to form N─H aziridines (Troc = ─COOCH_2_CCl_3_).

Trichloroethyl *N*‐tosyloxycarbamate **103** has been utilized as a nitrene precursor in the Cu‐catalyzed intermolecular aziridination of styrenes. The resulting Troc‐protected aziridines (i.e., **104**) could be hydrolyzed in the presence of LiOH to unveil the corresponding N─H aziridines **105** (Figure 46).^[^ ^158^ ^]^ The aziridination scope was limited to styrenes and the scope of deprotection was not fully examined (i.e., only two examples were described).

#### 3.1.3 Dealkylation of *N‐*Alkyl Aziridines

**Figure 47:**
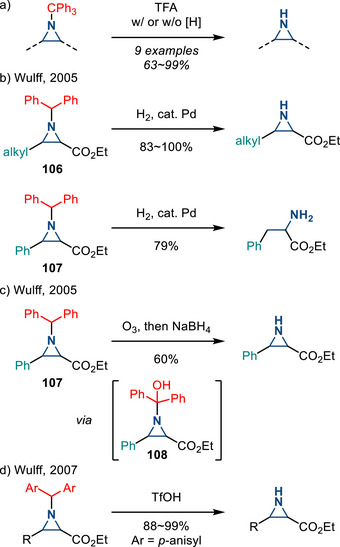
a) TFA‐promoted removal of *N‐*trityl substituents to form N─H aziridines. b) Substrate‐dependent dealkylation of aziridines via catalytic hydrogenation. c) Oxidative C─N cleavage via reductive ozonolysis to form N─H aziridine. d) Acid‐mediated dealkylation to form N─H aziridines (Ar = *p*‐anisyl).

*N*‐Alkyl aziridines can also serve as precursors to N─H aziridines. *N*‐Trityl substituents can be removed in the presence of trifluoroacetic acid (TFA) with or without a hydride reductant (Figure 47a).^[^ ^159^, ^160^, ^161^, ^162^ ^]^ Similarly, Wulff demonstrated deprotection of *N*‐benzhydryl (i.e., CHPh_2_) aziridines **106**, which were prepared by enantioselective carbene addition to the corresponding imines, under Pd‐catalyzed hydrogenolysis.^[^ ^163^ ^]^ These conditions were effective for 2‐alkyl aziridines, but ring‐opening was observed in the case of 2‐aryl aziridines (Figure 47b). To address this challenge, Wulff reported that *N*‐diphenylmethyl‐2‐aryl aziridines **107** could be deprotected in an ozonolysis‐reduction sequence. The authors speculated that initial oxygen‐atom transfer (OAT) generated aminal **108**, which was reductively cleaved to form the N─H aziridines (Figure 47c). In 2007, the same group demonstrated removal of dianisylmethyl in the presence of TfOH as a general deprotection strategy for both 2‐alkyl and 2‐aryl aziridines.^[^ ^164^ ^]^

**Figure 48:**
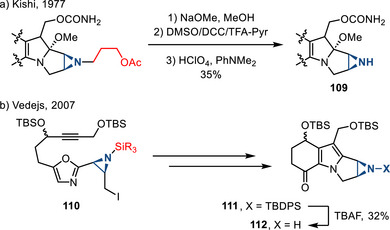
a) Deprotection of *N*‐(3‐acetoxy)propyl group to form N─H aziridines in the synthesis of mitomycins. b) Desilylation to form N─H aziridines in the construction of aziridinomitosene scaffolds.

In the context of complex molecule synthesis, challenges associated with ring‐opening and other decomposition reactions have given rise to bespoke *N*‐functionalization schemes. In Kishi's 1997 synthesis of mitomycins, the 3‐acetoxypropyl group was used as introduction of common *N*‐protecting groups proved inefficient. Three steps were involved in removal of the protecting group to form N─H aziridine **109** (Figure 48a).^[^ ^165^ ^]^ Similarly, Vedejs observed that aziridine deprotection of **110** was complicated. Therefore, he introduced *N‐*silyl groups to protect N─H aziridines during the synthesis of aziridinomitosene scaffolds. The *N‐*TBDPS protecting group survived relatively demanding oxazolium salt/azomethine ylide cycloaddition sequence used to construct the tricyclic core of the target (**111**). Treatment with TBAF afforded free N─H aziridines **112** (Figure 48b).^[^ ^166^ ^]^

#### 3.1.4 Removal of *N*‐Aryl Substituents

**Figure 49:**
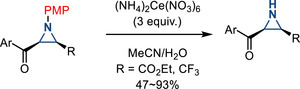
Oxidative dearylation of *N*‐*p*‐anisyl aziridines to form N─H aziridines using CAN.

Oxidative dearylation is another strategy used for *N*‐deprotection: *p*‐Anisidine derivatives have been reported to undergo oxidative dearylation with cerium(IV) ammonium nitrate (CAN). This method also works well with *N*‐PMP (*p*‐anisyl) aziridines to form N─H aziridines, which has been demonstrated by Akiyama, Carreria, Adrio, and Walsh (Figure 49).^[^ ^87^, ^167^, ^168^ ^]^ This dearylation strategy specifically requires the *para*‐methoxyphenyl *N*‐substituent, presumably due to its electron‐rich nature being susceptible to electron transfer under oxidative conditions.

#### 3.1.5 Removal of *N*‐Heterocyclic Substituents

**Figure 50:**
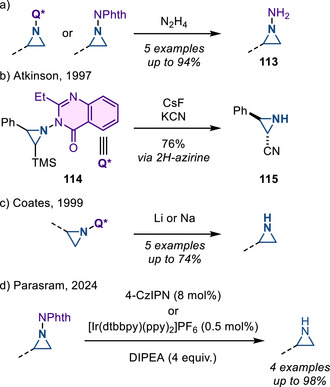
a) Hydrazinolysis of *N*‐phthalimido and *N*‐quinazolinonyl aziridines to form *N*‐amino aziridines. b) Aziridine functionalization to form 2‐cyanoaziridine. c) Reductive N─N cleavage of *N*‐quinazolinonyl aziridines to form N─H aziridines. d) Photoredox catalysis of *N*‐phthalimidoaziridines to form N─H aziridines.

*N*‐Aminophthalimide and 3‐amino‐2‐ethylquinazolin‐4(3*H*)‐one participate in olefin aziridination chemistry in the presence of Pb(OAc)_4_ or PhI(OAc)_2_. Hydrazinolysis of either gives rise to *N*‐aminoaziridines **113** (Figure 50a).^[^ ^169^, ^170^ ^]^ Atkinson reported a desilylation–cynation sequence of *N*‐quinazolinonyl aziridines **114** to form 2‐cyanoaziridines **115** via a 2*H*‐azirine intermediate (Figure 50b).^[^ ^171^ ^]^ Coates reported the reductive N─N cleavage of *N*‐quinazolinonyl aziridines with metal–ammonia or lithium–naphthalenide to form N─H aziridines (Figure 50c).^[^ ^172^ ^]^ Recently, Parasram reported the first example of N─N bond cleavage of *N*‐phthalimidoaziridines to form N─H aziridines under mild photoredox catalysis (Figure 50d).^[^ ^62^ ^]^ Although the mechanism has not yet been fully investigated, the deprotection likely occurs via reductive quenching of the excited photocatalyst, followed by single electron reduction of the phthalimido aziridine. While the reductive N─N cleaving deprotection of phthalimido aziridines has been reported with lithium, only one example was demonstrated to give the free N─H aziridine in 58% yield. This photoredox catalysis method extended the scope of phthalimido aziridine deprotection.

#### 3.1.6 Removal of Other *N*‐Substituents

**Figure 51:**
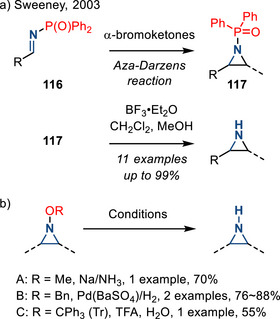
a) Dephosphinylation of *N*‐diphenylphosphinyl aziridines; b) removal of *N*‐oxy aziridines to form N─H aziridines.

*N*‐Diphenylphosphinyl aziridines **117**, which were prepared by aza‐Darzens reaction of the corresponding imine **116**, undergo dephosphinylation in the presence of BF_3_•Et_2_O (Figure 51a).^[^ ^173^ ^]^ *N*‐Oxy substituted aziridines are also common products of olefin aziridination reactions and a variety of conditions have been developed to achieve N─O cleavage and unveil N─H aziridines. Similar to sulfonylaziridine deprotection, N─OMe aziridines can be deprotected under Birch conditions.^[^ ^174^ ^]^ In addition to strongly reducing conditions, N─OBn substituents can be removed by Pd‐catalyzed hydrogenation, and *N*‐trityloxy groups can be removed by treatment with TFA (Figure 51b).^[^ ^175^ ^]^

### 3.2 *N‐*Functionalization of Aziridines

#### 3.2.1 Pd‐catalyzed N─H Functionalization

**Figure 52:**
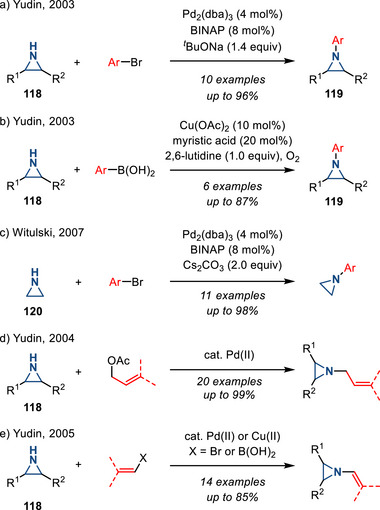
a) Pd‐catalyzed and b) Cu‐catalyzed *N*‐arylation of N─H aziridines. c) Pd‐catalyzed *N*‐arylation of ethyleneimine. d) Pd‐catalyzed *N*‐allylation of N─H aziridines. e) Pd‐ and Cu‐catalyzed alkenylation of N─H aziridines.

Pd‐catalyzed cross‐coupling of N─H aziridines can provide access to *N*‐allyl, *N*‐vinyl, and *N*‐aryl aziridines. In 2003, Yudin reported the Pd‐catalyzed coupling of N─H aziridines **118** with bromo(hetero)arenes to form *N*‐aryl aziridines **109** (Figure 52a).^[^ ^176^ ^]^ In the same report, the Cu‐catalyzed Chan‐Lam coupling of N─H aziridines with aryl boronic acids was also described (Figure 52b). For each reaction (i.e., Pd‐ and Cu‐catalyzed methods), only cyclohexene aziridine and 2‐benzoyl‐3‐*tert*‐butylaziridine were demonstrated. In 2007, Witulski et al. extended the substrate scope for N─H aziridine cross‐coupling to include ethyleneimine **120** (Figure 52c).^[^ ^177^ ^]^ Yudin et al. also developed allylation of N─H aziridines via a Pd‐catalyzed Tsuji–Trost type reaction with allyl acetates.^[^ ^178^, ^179^ ^]^ This allylation featured good regioselectivity and enantioselectivity when chiral BINAP ligand was incorporated (Figure 52d). Meanwhile, alkenylation of N─H aziridines was also reported via Pd‐ or Cu‐catalyzed cross‐coupling (Figure 52e).^[^ ^180^ ^]^ Seven and five aziridines were demonstrated for allylation and alkenylation, respectively.

#### 3.2.2 Direct N─N Bond Functionalization

**Figure 53:**
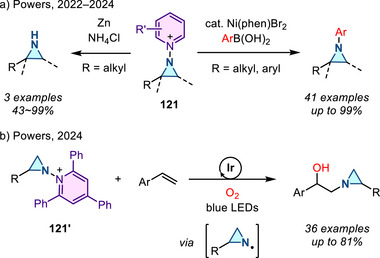
a) Derivatization of *N*‐pyridinium aziridines to form N─H and *N*‐aryl aziridines by reduction and nickel catalysis, respectively. b) Olefin 1,2‐hydroxyaziridination via aziridinyl radicals.

Recently, Powers demonstrated divergent *N*‐functionalization of *N*‐pyridinium aziridines. Pyridinium aziridines, prepared by olefin aziridination with *N*‐aminopyridinium salts, undergo Ni‐catalyzed cross‐coupling with boronic acid nucleophiles to yield *N*‐aryl aziridines.^[^ ^33^, ^34^ ^]^ This protocol bypasses the necessity for two‐step deprotection, *N*‐arylation sequences to access these structures. *N*‐Pyridinium aziridines **121** can also be converted to N─H aziridines under the action of Zn, though only three examples have been demonstrated (Figure 53a).^[^ ^34^ ^]^ The *N*‐pyridinium aziridines possess unique features compared to conventional protected aziridines because they readily generate aziridinyl radicals upon single electron reduction. This was demonstrated by the photocatalytic alkene hydroxyaziridination with pyridinium aziridines (Figure 53b).^[^ ^181^ ^]^

## 4 Biological Applications

The biological activity of aziridines can arise from either covalent or noncovalent interaction of the aziridine with biological targets.^[^ ^5^, ^10^, ^182^ ^]^ Covalent binding arises from ring‐opening alkylation reactions of biological nucleophiles, such as DNA and proteins.^[^ ^183^ ^]^ Despite the potential application of aziridines as covalent warheads, the application of aziridines in chemical biology is still underdeveloped. The lack of investigations may be due to the perception that these molecules display inherent cytotoxicity, although the cited literature relies on simple aziridines that lack molecular recognition domains and display nonspecific cytotoxicity.^[^ ^5^ ^]^ This section summarizes progress in aziridine‐based covalent inhibitors. Recent applications of aziridines in bioanalytical chemistry, particularly in the context of lipid characterization via mass spectrometry, are also discussed.

### 4.1 Covalent Protein Targeting of Aziridines

**Figure 54:**
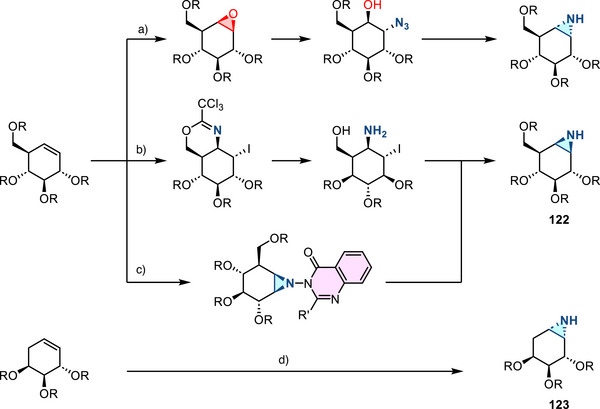
Synthetic strategies developed for the cyclophellitol aziridines (**122**–**123**). This family of aziridine‐based small molecules selectively binds aspartic acid and glutamic acid residues. Conditions a: i) *m*CPBA, ii) NaN_3_, H_2_O, iii) PPh_3_. b: i) CCl_3_CN, DBU, ii) I_2_, NaHCO_3_, H_2_O. c: i) 3‐amino‐2‐ethylquinazolin‐4(3*H*)‐one, PhI(OAc)_2_, K_2_CO_3_, ii) Na, NH_3_(l). d: DPH, Rh_2_(esp)_2_.

In the past decade, Overkleeft has studied the protein labeling of aziridines derived from cyclophellitol.^[^ ^184^, ^185^, ^186^ ^]^ The cyclophellitol aziridines are potent, mechanism‐based, irreversible inhibitors for glycoside hydrolases (vide infra). Figure 54 summarizes the synthetic chemistry of the cyclophellitol aziridines (**122**–**123**), which have been prepared via Routes A–D from the corresponding olefinic precursor.

#### 4.1.1 Glucosidase Inhibitors

**Figure 55:**
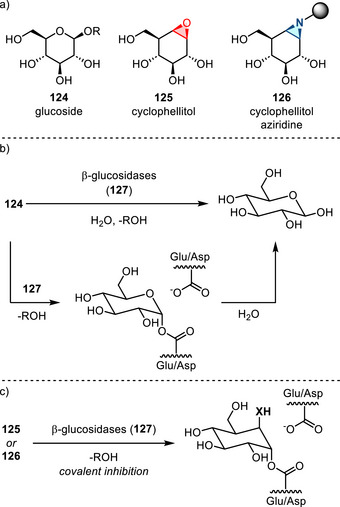
a) Structure mimics (**125**–**126**) of glucosides (**124**); b) Koshland double displacement mechanism of catalyzed glucoside hydrolysis; c) covalent inhibition of β‐glucosidases (**127**) using epoxides (**125**) and aziridines (**126**) (X = O or NR).

Cyclophellitol aziridines have been extensively studied as both inhibitors and activity‐based probes (ABP) for glycoside hydrolases and operate by covalent modification of the nucleophilic residues in the active site (Figure 55a): Retaining‐glycoside hydrolases (e.g., **127**) exploit a Koshland double displacement mechanism, where a carboxylate residue displacement, followed by water displacement, results in the hydrolyzed product retaining the stereochemistry (Figure 55b). Cyclophellitol aziridines **126** co‐opt this mechanism by engaging in nucleophilic ring‐opening with the carboxylate residue to form an aminoester, resulting in irreversible covalent inhibition of the hydrolase (Figure 55c).

**Figure 56:**
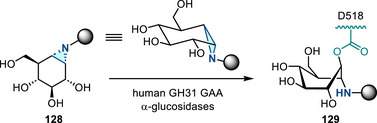
Aziridine **128** is a covalent inhibitor of α‐glucosidases via the formation of covalent adduct **129**.

Cyclophellitol aziridines (**126**) target β‐glucosidases (**127**) of various organisms (Figure 55c).^[^ ^187^, ^188^ ^]^ The identity of the exocyclic *N‐*substituents of **126** impacts the inhibitory activity: While *N*‐sulfonylation results in decreased activity compared to cyclophellitol, *N*‐alkylation results in 10–100‐fold more potency. X‐ray crystallography identified Glu‐441 as the nucleophilic site of human GBA2 enzyme covalently bound to aziridine **126**.^[^ ^189^ ^]^ Stereochemical modification at the aziridine resulted in ABPs targeting mammalian GH31 α‐glucosidases.^[^ ^190^ ^]^ The authors demonstrated in vitro inhibition and labeling of recombinant human GAA in the nanomolar range. To reveal the mode of action of **128**, a bacterial homologue of GAA was treated with aziridine **128**. X‐ray crystallography confirmed the covalent bonding with the enzymatic nucleophile site Asp‐412 (equivalent to Asp‐518 of human GAA), forming the covalent adduct **129** (Figure 56).

#### 4.1.2 Galactosidase and Mannosidase Inhibitors

**Figure 57:**
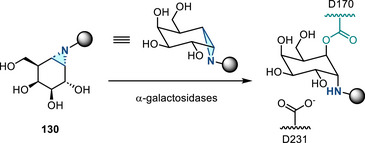
Covalent inhibition of α‐galactosidase.

In 2014, Overkleeft proposed the covalent binding between human GH27 α‐galactosidase (αGal A) with cyclophellitol aziridines **130**.^[^ ^191^ ^]^ To confirm the hypothesized inhibitory mechanism, the authors generated mutants lacking the active site nucleophile Asp‐170 or acid/base residue Asp‐231. The mutation of either residue led to the disappearance of inhibition with **130**. Thus, both the nucleophile and the acid/base residue were essential for covalent inhibition with aziridine small molecules (Figure 57).

**Figure 58:**
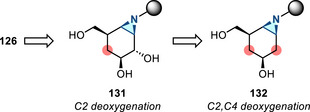
Deoxygenation at C2 and C4 positions of cyclophellitol aziridines.

In 2017, Overkleeft described efforts to develop broad‐spectrum glycoside probes (e.g., β‐glucosidases, β‐galactosidases, and β‐mannosidases). Since the carbohydrate substrate specificity of the glycoside hydrolase depends on the stereochemistry of C2 and C4, the authors hypothesized that deoxygenation of those positions (i.e., **131**–**132**, Figure 58) would provide inhibitory activities across different classes of glycoside hydrolases.^[^ ^192^ ^]^ Experimentally, **132** displayed labeling of purified β‐glucosidases and β‐galactosidases with low potency, and none of the probes showed activity toward β‐mannosidases.

**Figure 59:**
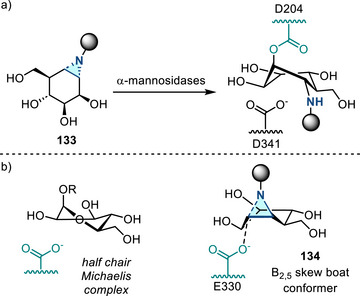
a) Covalent inhibition of α‐mannosidases with **133** through D204; b) substrate and high‐barrier transition state conformation for β‐mannosidases.

In 2020, Overkleeft reported low micromolar level inhibition of α‐mannosidases with aziridines **133**. Covalent liganding of Asp‐204, which is present in dGMII, was confirmed by X‐ray crystallography (Figure 59a).^[^ ^193^ ^]^ In comparison, analogous inhibition of β‐mannosidases with cyclophellitol scaffolds was less effective. This was attributed to conformational effects, which disfavored aziridine opening with the relevant nucleophile (Figure 59b). Nevertheless, covalent binding between cyclophellitol aziridine **134** and Glu‐330 of β‐mannosidase *Cm*Man5A was confirmed by X‐ray crystallography, as reported by Aerts and Davies in 2022.^[^ ^194^ ^]^

#### 4.1.3 Other Glycoside Hydrolase Inhibitors

**Figure 60:**
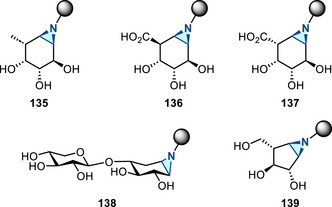
Cyclophellitol aziridines targeting other glycosidases.

Using similar strategies, Overkleeft and coworkers reported covalent inhibition of α‐fucosidase,^[^ ^195^ ^]^ β‐glucuronidase,^[^ ^196^ ^]^ α‐iduronidase,^[^ ^197^ ^]^ xylanase,^[^ ^198^ ^]^ and α‐arabinofuranosidase^[^ ^199^ ^]^ using corresponding cyclophellitol aziridines (**135**–**139**) illustrated in Figure 60.

**Figure 61:**
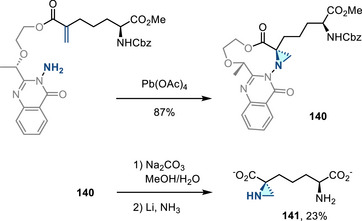
Stereoselective synthesis of Azi‐DAP (**141**) via intramolecular aziridination using tethered *N*‐amino quinazolinone.

Covalent binding between substrate‐mimicking aziridines and cysteine residues has also been reported. In 2005, Vederas et al. synthesized Azi‐DAP (diaminopimelic acid) (**141**) via intramolecular olefin aziridination using Pb(IV) as the terminal oxidant.^[^ ^200^ ^]^ The free N─H aziridine was obtained from reductive N─N bond cleavage in the presence of Li/NH_3_ (Figure 61). Azi‐DAP irreversibly binds DAP epimerase through Cys‐217, as confirmed by XRD. DAP epimerase participates in cell wall synthesis present in *Haemophilus influenzae*, and the inhibition of DAP epimerase potentially leads to antibiotic drug development against *H. influenzae*.

**Figure 62:**
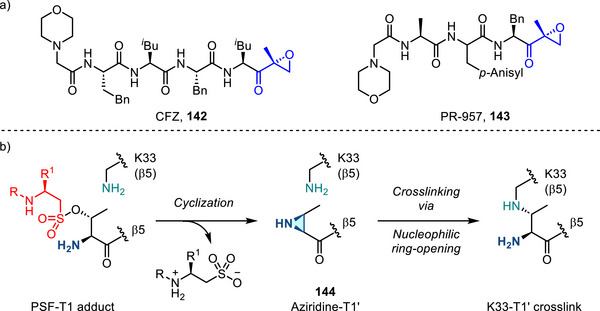
a) Inhibitors featuring α,β‐epoxyketone scaffold; b) aziridine‐T1’ as an intermediate to crosslink within proteosome.

Covalent binding of lysine residues with aziridines has also been reported in the context of immunoproteasome inhibition. The proteasome is a multicatalytic protease complex responsible for the ubiquitin‐dependent turnover of cellular proteins. Inhibition of proteasome results in cell death and has led to the development of targeted therapies to treat blood cancers (e.g., multiple myeloma and mantle cell lymphoma).^[^ ^201^ ^]^ Clinically applied inhibitors feature α,β‐epoxyketone present in CFZ (**142**) and PR‐957 (**143**) (Figure 62a).^[^ ^202^ ^]^ Selective binding of proteasome's subunit is crucial for the therapeutic window of the inhibitors. In 2014, Liskamp and Groll reported selective inhibition of immunoproteasome by crosslinking with aziridines. Aziridine intermediate **144** is generated in situ via *O‐*sulfonylation of the threonine residue (T1) within the β5 subunit of immunoproteasome, followed by cyclization.^[^ ^203^ ^]^ **144** then formed a cross‐link with the adjacent lysine residue (K33) via nucleophilic ring‐opening, as confirmed by mass spectrometry and X‐ray crystallography (Figure 62b). Notably, despite the inherent cytotoxic nature of aziridines, the authors observed low cytotoxicity of the precursor, potentially broadening the therapeutic window.

#### 4.1.4 Covalent Binding with KRAS Mutated Proteins

**Figure 63:**
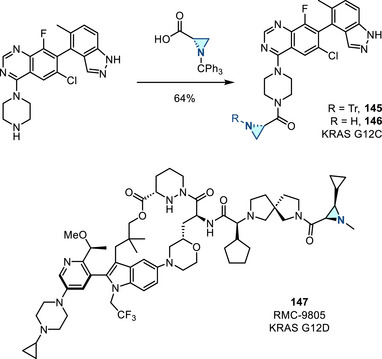
Aziridines targeting KRAS G12C and G12D.

Aziridines have also gained attention in selective binding with KRAS mutated proteins, which promote uncontrolled cell growth and potentially lead to cancer. Inhibition of such proteins could provide therapies against KRAS‐related cancer. In 2017, Shokat reported the aziridine **146** efficiently labeled KRAS G12C, as confirmed by LC‐MS and X‐ray crystallography. Aziridine **146** was prepared by dealkylation of *N*‐trityl aziridine precursor **145** (Figure 63). The α‐carbon of aziridine **146** was reactive toward Cys12 of KRAS G12C. Despite preferring small carboxylic acid substrates over thiols, aziridine **146** did not label G12D, likely due to the spatial arrangements of the electrophile.^[^ ^204^ ^]^ Recently, Revolution Medicines has developed an aziridine‐containing covalent tri‐complex inhibitor, RMC‐9805 (**147**), selectively targeting the G12D mutant to cyclophilin A and preventing binding to its canonical target RAF (Figure 63). Currently, RMC‐9805 is in a phase 1/1b clinical trial for the treatment of KRAS G12D mutant solid tumors.^[^ ^205^ ^]^

**Figure 64:**
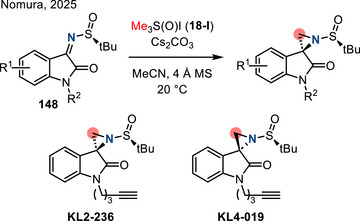
Stereoselective synthesis of aziridine via Corey–Chaykovsky reaction toward KL2‐236.

In 2025, Nomura evaluated a suite of stereochemically defined aziridines with *N‐*sulfinyl and *N*‐sulfonyl activating groups fused to a spirocyclic oxindole scaffold to map cryptic binding surfaces in the undruggable transcription factor, myelocytomatosis oncogene (MYC).^[^ ^206^ ^]^ The sulfinyl aziridines were prepared by diastereoselective aza‐Corey–Chaykovsky aziridination of ketimine precursors (**148**) (Figure 64). Notably, the study identified a covalent destabilizing MYC degrader, KL2‐236, whereas the diastereomer (KL4‐019) is largely inactive. KL2‐236 engages Cys‐203 and Asp‐205 residues in the intrinsically disordered region, highlighting that unstructured regions in high‐value proteins can be stereoselectively targeted.

**Figure 65:**
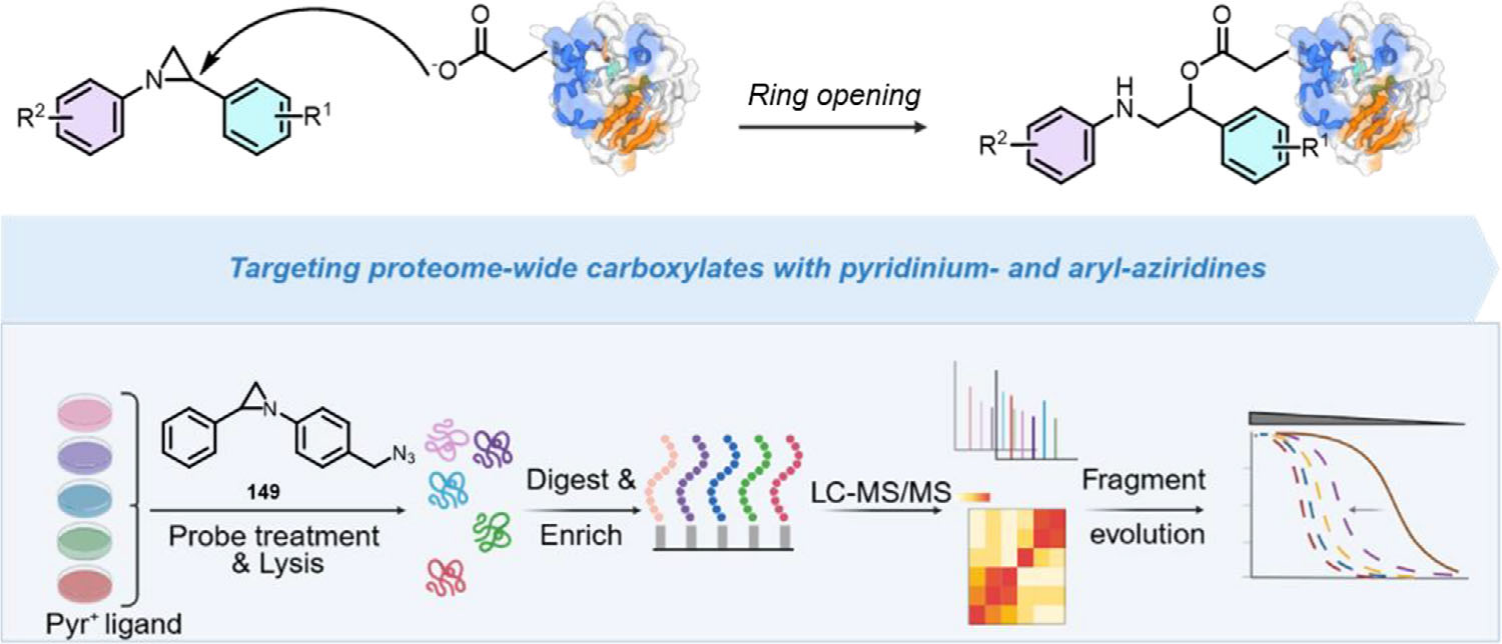
Schematic workflow of fragment screening, target identification, and fragment evolution to rapidly generate diverse *N*‐aryl aziridine‐based inhibitors.

In addition to a ligand‐first approach to generate mechanism‐based inhibitors, a target‐agnostic chemoproteomics approach enabled proteome‐wide screening of aziridine molecules to expand the ligandable space. In 2025, Powers and Adibekian identified *N*‐aryl aziridines (e.g., **149**) as a systematically tunable, chemoselective scaffold for covalent targeting of carboxylates across the proteome (Figure 65).^[^ ^207^ ^]^ Modular *build‐and‐couple* synthesis of *N*‐aryl aziridines from identified pyridinium aziridines enabled fragment evolution of aziridines with enhanced affinities for MTCH2 and RUFY1, which had not previously been targeted by small molecule inhibitors. This work established aziridines as carboxylate‐targeting covalent inhibitor candidates, broadening the scope of covalent ligand discovery.

### 4.2 Aziridines Applied to Lipidomics

Aziridination of C═C double bonds present in lipids has been advanced as a tool to identify lipid isomers by mass spectrometry analysis. Compared to epoxides, the aziridine derivatives are more ionizable, providing easier access to characteristic ion peaks during MS analysis.

**Figure 66:**
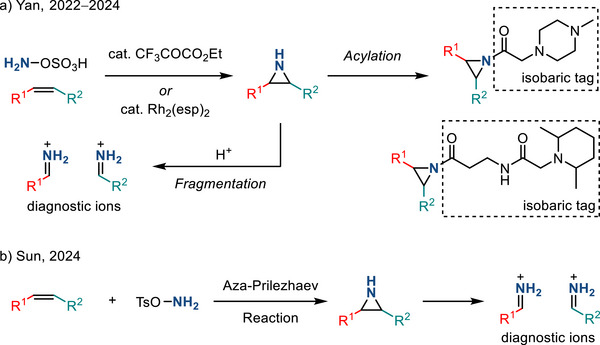
Aziridination of lipids with a) HOSA and b) TsONH_2_ to identify lipid isomers via mass spectrometry.

In 2022, Yan reported aziridination of C═C double bonds of lipids using HOSA as the aminating reagent.^[^ ^208^ ^]^ Aziridines subjected to tandem MS analysis underwent fragmentation to produce diagnostic ions, indicating the double bond position. The unsaturated sn‐positional isomers were also identified using this strategy. The N─H aziridines could also incorporate isobaric tags via acylation (Figure 66a), allowing for simultaneous quantification of analytes from multiple samples. Later in 2024, Yan applied this analytical method to colon cancer plasma, identifying 7 out of 17 lipid sn‐positional isomers with significant abundance change.^[^ ^209^ ^]^ A similar strategy was also demonstrated by Sun in 2024, adapting the aza‐Priezhaev reaction for lipid double bond aziridination. In this report, UMA‐MS was used to differentiate the *cis‐*/*trans*‐C═C double bond isomers (Figure 66b) (UMA‐MS = U‐shaped mobility analyzer‐mass spectrometry).^[^ ^210^ ^]^

**Figure 67:**
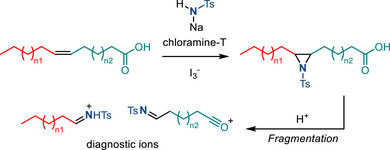
Aziridination with chloramine‐T for lipid analysis.

*N*‐Tosyl aziridines have also been reported in this context. Chloramine‐T can serve as a nitrene precursor in the preparation of N─Ts aziridines.^[^ ^211^ ^]^ In 2022, Sun, Zhang, and Guo applied the triiodide‐mediated olefin aziridination with chloramine‐T. The resulting N─Ts aziridines fragmented in MS to form diagnostic peaks to identify double bond positional isomers (Figure 67).^[^ ^212^ ^]^ Later in 2024, the *cis‐*/*trans*‐C═C double bond isomers were also differentiated by ion mobility MS, as reported by Guo and Wang.^[^ ^213^ ^]^

## 5 Conclusions and Outlook

The discovery of new synthetic methods for the construction of aziridines and broader appreciation for the need for methods that enable systematic control over the identity of the exocyclic nitrogen valence have garnered intense attention over the past decade. These synthetic innovations have paralleled and increased appreciation for the potential of aziridine‐containing small molecules in biological applications. In this review, we have summarized contemporary discoveries in aziridine synthesis, including transition‐metal‐ and metal‐free catalyzed methods, electrochemical synthesis, and light‐induced nitrene transfer. β‐C─H activation represents a newly appreciated strategy, and the biosynthetic equivalent has also been identified. The advances in aziridine synthesis are critical to realization of the potential bioactivity of aziridines and to the exhaustive evaluation of aziridine SAR. As a result, the removal and functionalization of the nitrogen valence were discussed. Following *N*‐functionalization, we highlighted aziridines’ relevance to medicinal chemistry, chemical biology, and analytical chemistry.

The importance of synthetic aziridine chemistry, both as a tool for small molecule synthesis and as an enabling platform for the development and optimization of bioactive small molecules, continues to stimulate intense interest and research activity. In just the past couple months, novel aziridination protocols based on iodonitrene intermediates and other electrophilic amine precursors have been disclosed.^[^ ^214^, ^215^ ^]^ These methods provide additional synthetic tools to address one of the central challenges in aziridine synthesis, namely, the development of general methods that enable facile preparation of specific *N*‐functionalized aziridines from broad and diverse families of starting materials.

Given the initial successes in 1) aziridination via β‐C─H activation, 2) photocatalytic desulfonylation of *N*‐sulfonyl aziridines, and 3) aziridine group transfer chemistry via aziridinyl radicals, we anticipate new synthetic methods will flourish in these (and many other) areas. With the development of modern synthetic technologies, such as electrochemistry, photocatalysis, and biocatalysis, more efficient and scalable methods for aziridine synthesis are expected to emerge. In return, biological applications of aziridines will benefit from the broadening chemical space of synthetically accessible aziridines. The intimate interplay of new synthetic tools and new biological applications represents exciting research opportunities and a call to arms for the continued development of general platforms for aziridine chemistry.

## Conflict of Interests

The authors declare no conflict of interest.

## Data Availability

Data sharing is not applicable to this article as no new data were created or analyzed in this study.
